# A weighted-sum chaotic sparrow search algorithm for interdisciplinary feature selection and data classification

**DOI:** 10.1038/s41598-023-38252-0

**Published:** 2023-08-28

**Authors:** LiYun Jia, Tao Wang, Ahmed G. Gad, Ahmed Salem

**Affiliations:** 1 Department of Mathematics and Physics, Hebei University of Architecture, Zhangjiakou, 075000 China; 2 Faculty of Computers and Information, Kafrelsheikh University, Kafrelsheikh, 33516 Egypt; 3 College of Computing and Information Technology, Arab Academy for Science, Technology and Maritime Transport (AASTMT), Cairo, Egypt

**Keywords:** Computer science, Computational science, Classification and taxonomy, Computational models, Information technology

## Abstract

In today’s data-driven digital culture, there is a critical demand for optimized solutions that essentially reduce operating expenses while attempting to increase productivity. The amount of memory and processing time that can be used to process enormous volumes of data are subject to a number of limitations. This would undoubtedly be more of a problem if a dataset contained redundant and uninteresting information. For instance, many datasets contain a number of non-informative features that primarily deceive a given classification algorithm. In order to tackle this, researchers have been developing a variety of feature selection (FS) techniques that aim to eliminate unnecessary information from the raw datasets before putting them in front of a machine learning (ML) algorithm. Meta-heuristic optimization algorithms are often a solid choice to solve NP-hard problems like FS. In this study, we present a wrapper FS technique based on the sparrow search algorithm (SSA), a type of meta-heuristic. SSA is a swarm intelligence (SI) method that stands out because of its quick convergence and improved stability. SSA does have some drawbacks, like lower swarm diversity and weak exploration ability in late iterations, like the majority of SI algorithms. So, using ten chaotic maps, we try to ameliorate SSA in three ways: (i) the initial swarm generation; (ii) the substitution of two random variables in SSA; and (iii) clamping the sparrows crossing the search range. As a result, we get CSSA, a chaotic form of SSA. Extensive comparisons show CSSA to be superior in terms of swarm diversity and convergence speed in solving various representative functions from the Institute of Electrical and Electronics Engineers (IEEE) Congress on Evolutionary Computation (CEC) benchmark set. Furthermore, experimental analysis of CSSA on eighteen interdisciplinary, multi-scale ML datasets from the University of California Irvine (UCI) data repository, as well as three high-dimensional microarray datasets, demonstrates that CSSA outperforms twelve state-of-the-art algorithms in a classification task based on FS discipline. Finally, a 5%-significance-level statistical post-hoc analysis based on Wilcoxon’s signed-rank test, Friedman’s rank test, and Nemenyi’s test confirms CSSA’s significance in terms of overall fitness, classification accuracy, selected feature size, computational time, convergence trace, and stability.

## Introduction

The twenty-first century has become the era of data, with data analysis and utilization visible everywhere in all aspects of life, and these data are frequently of high-dimensional character^1–5^. However, it is inevitable that this data will contain a substantial number of redundant and irrelevant characteristics, increasing the computational overhead and risk of overfitting when handled by traditional machine learning (ML) algorithms^6–8^. As a result, in order to make better use of the data, efficient procedures, such as feature selection (FS), must be developed to handle the worthless features^9–11^. Wrappers, filters, and embedded FS techniques are commonly used to differentiate them based on their evaluation for feature subsets^12^. Wrapper-based approaches rely on predefined ML algorithms to obtain higher classification accuracy but are very expensive to compute because the ML algorithms must be run numerous times^13^. On the contrary, while evaluating feature subsets, filter-based approaches do not use any ML algorithms, which reduces computing cost but may reduce classification accuracy^14^. Embedded techniques incorporate FS into model learning, accounting for the influence of the algorithmic model while lowering computational weight; however, these methods have poor generalization ability and significant computational complexity^15^.

Because the number of feature subsets varies geometrically due to data dimensionality, it is challenging to produce adequate results using traditional methods, especially when working on high-dimensional data. To reduce the high computational cost caused by the curse of dimensionality, novel feature subset selection approaches can be developed based on wrapper swarm intelligence (SI) algorithms due to their robustness and adjustability^16–18^. SI algorithms have three essential characteristics: flexibility, self-organization, and resilience. These algorithms are often inspired by group behavior in nature, such as foraging, anti-predation, and migration^19^. Typical SI algorithms are ant colony optimization (ACO)^20^, particle swarm optimization (PSO)^21^, grey wolf optimizer (GWO)^22^, artificial bee colony (ABC)^23^, whale optimization algorithm (WOA)^24^, grasshopper optimization algorithm (GOA)^25^, harris hawks optimization (HHO)^26^, and bird swarm algorithm (BSA)^27^. Other optimization algorithms include bat algorithm (BA)^28^, atom search optimization (ASO)^29^, and henry gas solubility optimization (HGSO)^30^. In general, meta-heuristic algorithms can effectively handle FS problems, lowering computational complexity while achieving a greater classification accuracy, and SI approaches have, therefore, been consistently applied to FS problems^31–34^. For instance, Hussain et al.^35^ integrated the sine-cosine algorithm (SCA) into HHO to balance the exploration and exploitation capabilities of HHO, and experimental results on several numerical optimization as well as FS problems revealed the competitive advantage of the proposed algorithm over other SI algorithms. Neggaz et al.^36^ first applied HGSO to solving FS problems. Experimental results on datasets with different feature sizes (from 13 to 15009) showed that HGSO is effective in minimizing feature size, especially on high-dimensional data, while preserving maximum classification accuracy.

Nevertheless, SI algorithms tend to fall into local optimization due to: (i) the imbalance between exploration and exploitation; and (ii) super stochasticity^37,38^. Numerous studies have shown that chaos theory can defeat such an issue owing to its characteristics of semi-stochastic, ergodicity, and sensitivity to the initial swarm^39,40^. Khosravi et al.^41^ incorporated a new local search strategy and the Piecewise chaotic map, in order to make their teaching optimization algorithm capable of tackling high-dimensional FS problems. Zhang et al.^42^ integrated the Gaussian’s mutation and the Logistic chaotic map into the fruit fly algorithm (FFA) to avoid premature convergence and hence strengthen the exploration capability. Sayed et al.^43^ optimized the crow search algorithm (CSA) by using ten chaotic maps to improve its performance in tackling FS problems in terms of classification accuracy, number of selected features, and convergence speed. Altay et al.^44^ replaced the random parameters in BSA with ten chaotic maps to boost the exploration ability.

The sparrow search algorithm (SSA) is one of many recently developed SI algorithms. In it, the sparrow is a dexterous species that forages through collective collaboration and can effectively escape natural predators. SSA was proposed by Xue et al.^45^ by emulating such properties. When compared to its counterparts, SSA has garnered a lot of attention because of its fast convergence, great search efficiency, and stability^46–51^. However, SSA suffers from the same flaws as other SI algorithms in that swarm diversity and exploratory abilities decrease as the algorithm progresses^47,52^. As a result, significant enhancements have been made to SSA. To make SSA more thorough in exploring the solution space, Xue et al.^53^ utilized a new neighbor search approach. Gao et al.^52^ added a chaotic map and a mutation evolution technique to SSA to improve its robustness and convergence speed. Gad et al.^54^ binarized SSA using S- and V-shaped functions and included a random relocation approach for transgressive sparrows as well as a new local search strategy to balance its exploration and exploitation capabilities. Lyu et al.^55^ used the Tent chaotic map and the Gaussian mutation technique to improve SSA and apply it to simple image segmentation challenges. Furthermore, Yang et al.^56^ improved SSA with the use of the Sine chaotic map, an adaptive weighting approach, and an adaptive t-distribution mutation operator, and then applied the suggested technique to numerical optimization problems. However, no one has yet used a chaos-improved SSA to solve FS problems. SI algorithm performance can generally be improved in three ways: (i) adjusting their parameters; (ii) altering their mechanisms; and (iii) combining them with other algorithms^57^. In light of this, this work aims to improve SSA by redefining its random parameters and procedures through the use of a chaotic map. The following are the main contributions:

1. The initial swarm, transgressive positions, and random variables in SSA are processed by using chaotic maps to simultaneously boost its swarm diversity and make a good trade-off between exploration and exploitation in it. Comparing twenty different chaos-improved SSA variants yields the best chaotic SSA (CSSA).
2. CSSA is compared against twelve peer algorithms, including SSA, ABC, PSO, BA, WOA, GOA, HHO, BSA, ASO, HGSO, success-history based adaptive differential evolution with linear population size reduction (LSHADE)^58^ and evolution strategy with covariance matrix adaptation (CMAES)^59^, on some representative functions from the Institute of Electrical and Electronics Engineers (IEEE) Congress on Evolutionary Computation (CEC) and eighteen multi-scale datasets from the University of California Irvine (UCI) data repository as a scaffold to verify its competitiveness. Furthermore, this study also selects seven recently proposed FS methods from the literature to verify that CSSA still has advantages over several state-of-the-art algorithms.
3. The capability of CSSA is further tested on three high-dimensional microarray datasets with a number of features/genes (dimensions) up to 12500.
4. We empirically and theoretically measure the strengths and weaknesses of CSSA against different algorithms to solve FS problems under evaluation metrics, such as overall fitness, classification accuracy, selected feature size, convergence, and stability.
5. A post-hoc statistical analysis, including Wilcoxon’s signed-rank test, Friedman’s rank test, and Nemenyi’s test, is conducted at a 5% significance level to verify the statistical significance of CSSA over its peers.

Following that, this article is organized as follows. Section Preliminaries introduces the SSA principle and the ten chaotic maps that have been tested with it, whereas Sect. Proposed chaotic sparrow search algorithm (CSSA) presents the proposed CSSA. Section Experimental results and discussion compares CSSA to twelve peer algorithms and seven popular FS approaches in the literature, and experimental data on eighteen UCI datasets and three high-dimensional microarray datasets are provided and analyzed. Section Discussion discusses CSSA’s strengths and limitations. Finally, Sect. Conclusion concludes the paper.

## Preliminaries

### Sparrow search algorithm (SSA)

This section presents a brief history of SSA and its mathematical formulation. SSA is a recently developed SI algorithm that in a mathematical language mimics the foraging and anti-predatory behaviors of sparrows. In general, sparrows are classed as producers or scroungers based on their fitness values, which are assessed on a regular basis using individuals’ current positions. Producers are largely responsible for supplying food to the swarm, whereas scroungers often use producers as a means to get a source of food. In addition, as predators approach the swarm, some scouters modify their positions to protect themselves and the entire swarm. As a result, the sparrow swarm can continuously gather food while also ensuring security for the swarm’s reproduction under various strategies. Different species of sparrows have different roles, and the following are the components of SSA and its algorithmic process.

***Step 1***: The swarm is initialized. SSA first randomly generates the initial positions of a group of sparrows as
  1 $$\begin{aligned} {\textbf{X}}=\left[ \begin{array}{c} {\textbf{x}}_{1} \\ {\textbf{x}}_{2}\\ \vdots \\ {\textbf{x}}_{N} \end{array}\right] = \left[ \begin{array}{cccc}x_{1,1} &{} x_{1,2} &{} \ldots &{} x_{1, D} \\ x_{2,1} &{} x_{2,2} &{} \ldots &{} x_{2, D} \\ \vdots &{} \vdots &{} \ldots &{} \vdots \\ x_{N, 1} &{} x_{N, 2} &{} \ldots &{} x_{N, D}\end{array}\right] \text {, } x_{i,j} \sim U{(0,1)}, \end{aligned}$$
  where *N* denotes the number of individuals in the swarm, *D* represents the dimensionality of a decision vector (or the number of features in a dataset being processed in the case of FS problems), and $x_{i,j}$ denotes a value taken by a sparrow *i* in a dimension *j*. SSA judges the quality of obtained solutions via a fitness function
  2 $$\begin{aligned} \mathbf {F({\textbf{X}})}=\left[ \begin{array}{c}f({\textbf{x}}_{1}) \\ f({\textbf{x}}_{2}) \\ \vdots \\ f({\textbf{x}}_{N}) \end{array}\right] , \end{aligned}$$
  where a fitness function *f*(.) is used to evaluate the quality of a given solution ${\textbf{x}}_i$.
***Step 2***: The producer is mainly responsible for finding food sources, and its position update rules are
  3 $$\begin{aligned} {\textbf{x}}_i^{t+1}=\left\{ \begin{array}{lll}{\textbf{x}}_i^{t} \exp \left( \frac{-i}{\alpha T}\right) , &{} \text{ if } &{} R_{2}<ST\\ {\textbf{x}}_i^{t}+QL, &{} \text{ if } &{} R_{2} \ge ST\end{array}\right. \end{aligned}.$$
  SSA improves the quality of its solutions by exchanging information among its consecutive iterations. Eq. (3) is used to describe the way information is exchanged between producers as the number of iterations increases. *t* denotes current iteration’s number. Since SSA is not used to find the global optimal solution, but to provide a relatively better solution, the maximum number of iterations *T* is usually used as the condition for the termination of the algorithm. $\alpha$ usually has a random value in the range [0, 1]. The warning value $R_2 \sim U{(0,1)}$ indicates the hazard level of a producer’s location, while the safety value $ST \in [0.5,1]$ is a threshold value used to determine whether a producer’s location is safe. $R_2<ST$ indicates that the producer is in a safe environment and can search extensively; otherwise, the producer is at risky location of predation and needs to fly away. *Q* is a random parameter that follows a normal distribution. *L* denotes a $1 \times D$ matrix with all its elements having values equal to 1.
***Step 3***: The swarm in SSA can be divided into producers and scroungers. The scroungers renew themselves as
  4 $$\begin{aligned} {\textbf{x}}_i^{t+1}=\left\{ \begin{array}{lll}Q \exp \left( \frac{{\mathbf{g}}_{worst}^{t}-{\textbf{x}}_i^{t}}{i^2}\right) , &{} \text { if } i>N / 2 \\ {\mathbf{g}}_{best}^{t+1}+|{\textbf{x}}_i^{t}-{\mathbf{g}}_{best}^{t+1}|A^{+}L, &{} \text { otherwise }\end{array}\right. \end{aligned},$$
  where ${\mathbf{g}}_{worst}$ and ${\mathbf{g}}_{best}$ denote the current global worst and best positions, respectively, with the help of which the discoverers can improve the convergence speed of the algorithm, but it increases the risk of falling into a local optimum. $A^+=A^T(AA^T)^{-1}$, where *A* denotes a $1 \times D$ matrix with each element in it having a value randomly set to 1 or $-1$. Eq. (4) shows that $i>N/2$ indicates that scroungers need to fly elsewhere to get food; otherwise, scroungers get food form around producers.
***Step 4***: Scouters are randomly selected from the swarm, typically 10–20% of the total swarm size, and they are updated as
  5 $$\begin{aligned} {\textbf{x}}_i^{t+1}=\left\{ \begin{array}{ll}{\mathbf{g}}_{worst}^{t}+\beta |{\textbf{x}}_i^{t}+{\mathbf{g}}_{best}^{t}|, &{} \text{ if } f({\textbf{x}}_i^{t})>f({\mathbf{g}}_{best}^{t}) \\ {\textbf{x}}_i^{t}+K\left( \frac{|{\textbf{x}}_i^t+{\mathbf{g}}_{worst}^{t} |}{|f({\textbf{x}}_i^{t})-f({\mathbf{g}}_{worst}^{t})|+\sigma }\right) , &{} \text{ if } f({\textbf{x}}_i^{t})=f({\mathbf{g}}_{best}^{t})\end{array}\right. \end{aligned},$$
  where $\beta$ takes a random value with normal distribution properties, *K* is a parameter that takes a random value between $-1$ and 1, $\sigma$ is a constant to avoid the occurrence of an error when the denominator is 0, and $f({\mathbf{g}}_{best}^{t})$ and $f({\mathbf{g}}_{worst}^{t})$ are fitness values of the current global best and worst individuals, respectively. The scouters take fitness according to an update criterion, i.e., $f({\textbf{x}}_i^{t})>f({\mathbf{g}}_{best}^{t})$ indicates that the sparrow is at risk of predation and needs to change its location according to the current best individual, whereas when $f({\textbf{x}}_i^{t})=f({\mathbf{g}}_{best}^{t})$, a sparrow needs to strategically move closer to other safe individuals to improve its safety index.
***Step 5***: Updation and stopping guidelines are applied. The current position of a sparrow is only updated if its corresponding fitness is better than that of previous position. If the maximum number of current iteration is not reached, then return to ***Step 2***; otherwise, output position and fitness of the best individual.

Thus, the basic framework of SSA is realized in Algorithm 1.

**Figure:**
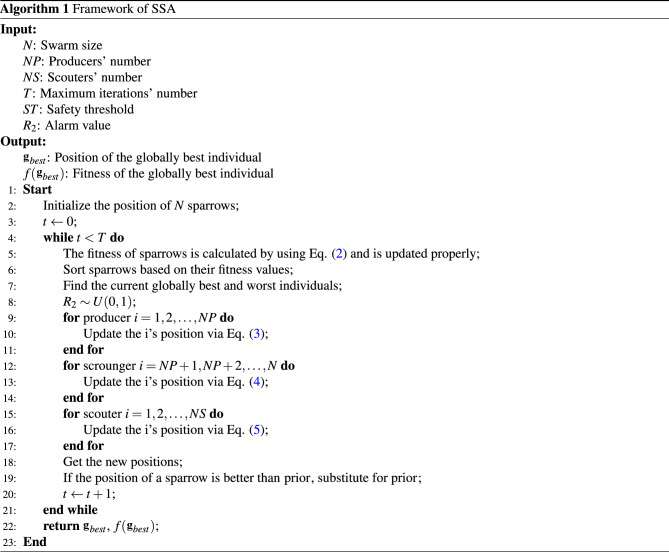

### Chaotic maps

**Figure 1:**
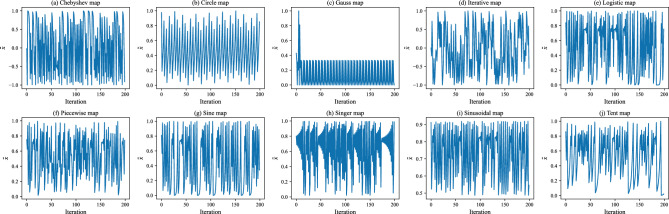
Visualizations of the ten chaotic maps used in this study and generated by using Matplotlib 3.5.2^61^ in Python 3.9.12^62^.

Chaos is defined as a phenomenon and exhibits some sort of chaotic behavior by using an evolution function and have three main characteristics: i) quasi-stochastic; ii) ergodicity; and iii) sensitivity to initial conditions^60^. If its initial condition is changed, this may lead to a non-linear change in its future behavior. Thus, stochastic parameters in most algorithms can be strengthened by using chaos theory, given that the ergodicity of chaos can help explore the solution space more fully. Table 1 presents the mathematical expressions for the ten chaotic maps used in this study^44^, where ${\tilde{x}}$ represents the random number generated from a one-dimensional chaotic map. Figure 1 shows their own visualizations, as well.

**Table 1:** Definition of the ten chaotic maps used in this study.

Name	Definition	Condition	Range
Chebyshev map	${\tilde{x}}^{t+1}=\cos (k \cos ^{-1}({\tilde{x}}^{t}))$	$k=2$	$[-1,1]$
Circle map	${\tilde{x}}^{t+1}={\tilde{x}}^{t}+b-\left( \frac{a}{2 \pi }\right) \sin (2 \pi {\tilde{x}}^{t}) \bmod (1)$	$a=0.5$ and $b=0.2$	[0, 1]
Gauss map	$${\tilde{x}}^{t+1}= {\left\{ \begin{array}{ll}0, &{} \text { if } x=0 \\ \frac{1}{{\tilde{x}}^{t} \bmod (1)}=\frac{1}{{\tilde{x}}^{t}}-\left[ \frac{1}{{\tilde{x}}^{t}}\right] , &{} \text { if } {\tilde{x}}^{t} \in ]0,1]\end{array}\right. }$$	–	[0, 1]
Iterative map	${\tilde{x}}^{t+1}=\sin \left( \frac{a \pi }{{\tilde{x}}^{t}}\right)$	$a=0.7$	$[-1,1]$
Logistic map	${\tilde{x}}^{t+1}=a {\tilde{x}}^{t}(1-{\tilde{x}}^{t})$	$a=4$	[0, 1]
Piecewise map	$${\tilde{x}}^{t+1}= {\left\{ \begin{array}{ll}\frac{{\tilde{x}}^{t}}{P}, &{} \text { if } 0 \le {\tilde{x}}^{t}<P \\ \frac{{\tilde{x}}^{t}-P}{0.5-P}, &{} \text { if } P \le {\tilde{x}}^{t}<0.5 \\ \frac{1-P-{\tilde{x}}^{t}}{0.5-P}, &{} \text { if } 0.5 \le {\tilde{x}}^{t}<1-P \\ \frac{1-{\tilde{x}}^{t}}{P}, &{} \text { if } 1-P \le {\tilde{x}}^{t}<1\end{array}\right. }$$	$P=0.4$	[0, 1]
Sine map	${\tilde{x}}^{t+1}=\frac{a}{4} \sin \left( \pi {\tilde{x}}^{t}\right)$	$a=4$	[0, 1]
Singer map	${\tilde{x}}^{t+1}=\mu (7.86 {\tilde{x}}^{t}-23.31 {\tilde{x}}^{t^2}+28.75 {\tilde{x}}^{t^3}-13.302875 {\tilde{x}}^{t^4})$	$\mu =1.07$	[0, 1]
Sinusoidal map	${\tilde{x}}^{t+1}=a {\tilde{x}}^{t^2} \sin (\pi {\tilde{x}}^{t})$	$a=2.3$	[0, 1]
Tent map	$${\tilde{x}}^{t+1}= {\left\{ \begin{array}{ll}\frac{{\tilde{x}}^{t}}{0.7}, &{} \text { if } {\tilde{x}}^{t}<0.7 \\ \frac{10}{3 {\tilde{x}}^{t}(1-{\tilde{x}}^{t})}, &{} \text{ otherwise } \\ \end{array}\right. }$$	–	[0, 1]

## Proposed chaotic sparrow search algorithm (CSSA)

In this study, CSSA is produced by mitigating the deficiencies of SSA through chaotic maps in three aspects: i) initial swarm; ii) two random parameters; and iii) clamping the sparrows crossing the search space. The initial swarm of SSA is usually generated randomly, and swarm diversity is thus easily eventually lost, leading to a lack of extensive exploration of the solution space. This can be regularly amended throughout the iterative process by utilizing the ergodic nature of chaos. For the two random parameters, this study considers $\alpha$ in the producer (Eq. (3)) and *K* in the scouter (Eq. (5)). Since $\alpha \in [0,1]$, it is replaced clearly by any of the ten chaotic maps, conditioned that the Chebyshev and Iterative chaotic maps take absolute values. Also, $K \in [-1,1]$, so this study finally settles its replacement with the Chebyshev map. Finally, the position of sparrows going outside the search range is also clamped with the help of chaotic maps by redefining it as

6 $$\begin{aligned} x_{i,j}^{t}=\left\{ \begin{array}{ll} x_{i,j}^{t}, &{} \text { if } x_{i,j}^{t} \in [0,1] \\ {\tilde{x}}_{i,j}^{t}, &{} \text { otherwise }\end{array},\right. \end{aligned}$$

where $x_{i,j}^{t}$ and ${\tilde{x}}_{i,j}^{t}$, respectively, represent the original and chaotic positions of a sparrow *i* at a dimension *j* and an iteration *t*. By analyzing the experimental results in Section Comparative analysis, the final version of CSSA is eventually released with the following final configuration: (i) the Circle map is used to generate the initial swarm, replace $\alpha$ in Eq. (3), and relocate the sparrows crossing the search range via Eq. (6); and (ii) the Chebyshev map substitutes for *K* in Eq. (5).

Only using the best individuals in SSA to guide the evolutionary direction of its swarm improves its convergence speed but also increases the risk of falling into a local optimum. To address this issue, SSA sets some random numbers in the algorithm, but the random number generator used is not without sequential correlation in successive calls, so swarm diversity still decreases in the late iteration of the algorithm. The randomness and unpredictability of chaotic sequences can be then utilized in the generation of random numbers to enhance swarm diversity of SSA, thus increasing its exploration capability to scrutinize the search space more widely^63,64^. Thus, this work uses chaotic maps to generate the initial swarm of SSA and replaces some random numbers in it.

### Solution encoding

To our knowledge, binary vectors^65^ are substantial to encode features in FS problems, and a facilitative scheme (e.g., transfer functions) can be used to convert the continuous search space into a binary one^66^, in which 0s and 1s are used to organize the position of individuals. All features are initially selected, and during subsequent iterations, a feature is denoted as 1 if it is selected; otherwise, it is represented as 0. In this study, to construct the binary search space, CSSA is discretized by using a V-shaped transfer function^67^ as

7 $$\begin{aligned} V\left( {\textbf{x}}_i^{t+1}\right) =\left|\frac{2}{\pi } \arctan \left( \frac{\pi }{2} {\textbf{x}}_i^{t+1}\right) \right|. \end{aligned}$$

Thus, the locations of SSA’s individuals are made up of binary vectors^68^ as

8 $$\begin{aligned} x_{i,j}^{t+1}={\left\{ \begin{array}{ll}\lnot { x_{i,j}^{t} }, &{} \text { if } r < V\left( x_{i,j}^{t+1}\right) \\ x_{i,j}^{t}, &{} \text { otherwise }\end{array}\right. }, \end{aligned}$$

where $r \sim U{(0,1)}$. $r < V(\cdot)$ means that if a feature is previously selected, it is now discarded and vise versa; otherwise, a feature’s selection state is preserved.

### Flow of CSSA

CSSA first builds an initial swarm using chaotic maps. Depending on the range of the chaotic maps, the initial point of the chaotic maps can take any value between 0 and 1, for example, the initial point of the Chebyshev and Iterative chaotic maps can take a value between −1 and 1. An initial value ${\tilde{x}}^{0}$ for a chaotic map may have a significant influence of fluctuation patterns on it. So, except for the Tent chaotic map where ${\tilde{x}}^{0}=0.6$, we utilize ${\tilde{x}}^{0}= 0.7$^43,69^ for all chaotic maps. Each location of a sparrow represents a possibly viable solution conditioned by clamping inside the range [0, 1] for each of its dimensions.

Second, a determinant is required to assess the quality of each binarized solution we obtain. FS problems typically include two mutually exclusive optimization objectives, namely, maximizing classification accuracy and lowering selected feature size. Weighted-sum methods are extensively employed in this type of problem due to their straightforwardness and simplicity of implementation^70^. We employ the weighted-sum approach in the fitness function to achieve a good trade-off between the two objectives as

9 $$\begin{aligned} Fit_i = \gamma Err_i + (1-\gamma ) \frac{\vert S_i\vert }{D}, \end{aligned}$$

where *k*-Nearest Neighbor (*k*-NN, $k=5$^31,54^) and $Err_i$ represent the classification algorithm that is run on selected features in a solution *i* and the respective classification error rate, respectively. *k*-NN is commonly used in combination with meta-heuristics in classification tasks for solving FS problems due to its computational efficiency^54^. $\vert S_i\vert$ represent the number of useful features CSSA has selected in *i*. A smaller feature selection ratio indicates that the algorithm has more effectively selected useful features. $\gamma$ represents a weighting coefficient, which is set to 0.99 according to existing studies^54,71^.

Next, the position of sparrows is updated according to Eqs. (3), (4), and (5), provided that $\alpha$ and *K* are replaced with independent random values generated by a given chaotic map. This highly support the search agents of CCSA to more effectively explore and exploit each potential region of the search space.

**Figure 2:**
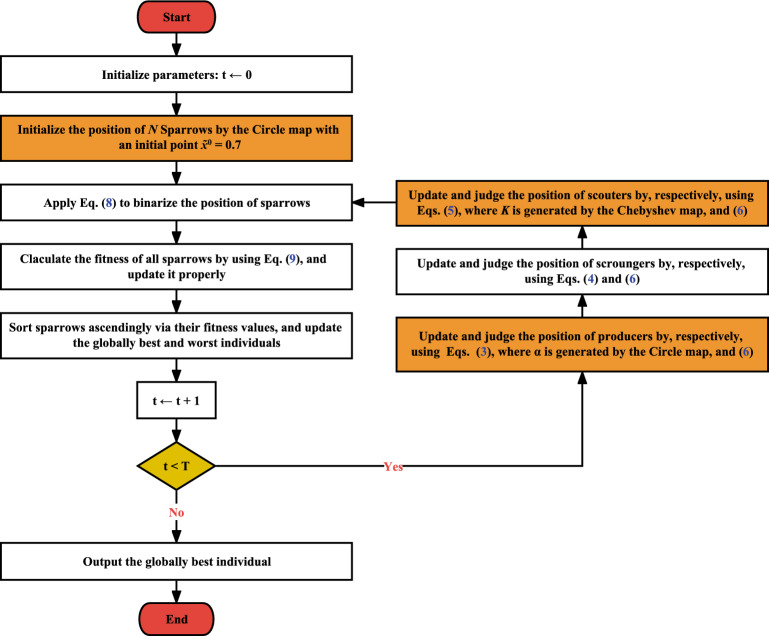
Flowchart of CSSA.

Finally, CSSA terminates based on a predefined termination condition. For optimization problems, there are typically three termination conditions: (i) the maximum number of iterations is reached; (ii) a decent solution is obtained; and (iii) a predetermined time window. The first condition is used as the termination condition in this study. Overall, CSSA is realized in Algorithm 2. For the sake of simplicity, Fig. 2 depicts its flowchart, as well.

**Figure:**
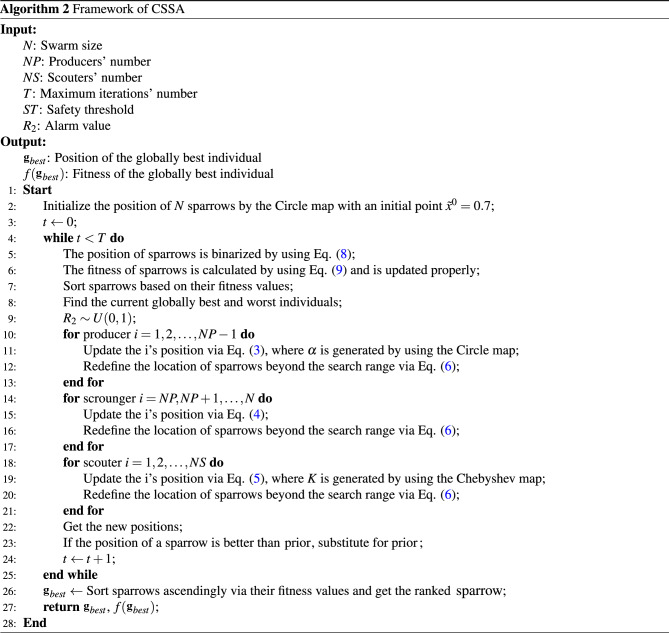

### Computational complexity analysis

Feature selection based on wrapper methods evaluates the candidate subsets several times in the process of finding the optimal feature subset, which increases the complexity of the algorithm. Therefore, this section analyzes the overall complexity of CSSA in the worst case.

To facilitate the analysis of CSSA’s time complexity, Algorithm 2 is inspected step by step. In the initialization phase (Line 2), the position of *N* sparrows is initialized with $\mathcal{O}$(*N*) time complexity. In the main loop phase, the time complexity of binarization (Line 5), solution evaluation (Line 6), and updating positions and redefining variables going outside the bounds (Lines 10–21) is $\mathcal{O}$(*N*), $\mathcal{O}(N+N \log {N}+1)$, and $\mathcal{O}$(2*N*), respectively. Finally, finding the globally best individual (Line 6) has a time complexity of $\mathcal{O}(\log {N})$. Thus, the worst time complexity of CSSA can be defined as $\mathcal{O}(N)+\mathcal{O}(T((N+N+N \log {N}+1)+2N))+\mathcal{O}(\log {N})=\mathcal{O}(N)+\mathcal{O}(T(4N+N \log {N}+1))+\mathcal{O}(\log {N})=\mathcal{O}(TN \log {N})$. On the other hand, the space complexity of CSSA is measured by overhead imposed by it on memory, i.e., $\mathcal{O}$(*ND*).

## Experimental results and discussion

### Dataset description

In this study, experiments are conducted on eighteen UCI datasets listed in Table 2, covering different subject areas, including physics, chemistry, biology, medicine, etc^72^. Interdisciplinary datasets have advantages to evaluate the applicability of CSSA in multiple disciplines.

**Table 2:** Characteristics of eighteen UCI datasets.

Dataset	No. of features	No. of instances	No. of classes	Domain
Breastcancer	9	699	2	Biology
BreastEW	30	569	2	Biology
CongressEW	16	435	2	Politics
Exactly	13	1000	2	Biology
Exactly2	13	1000	2	Biology
HeartEW	13	270	2	Biology
IonosphereEW	34	351	2	Electromagnetic
KrvskpEW	36	3196	2	Game
Lymphography	18	148	2	Biology
M-of-n	13	1000	2	Biology
PenglungEW	325	73	7	Biology
SonarEW	60	208	2	Biology
SpectEW	22	267	2	Biology
Tic-tac-toe	9	958	2	Game
Vote	16	300	2	Politics
WaveformEW	40	5000	3	Physics
WineEW	13	178	3	Chemistry
Zoo	16	101	7	Artificial

### Performance metrics

We mainly use four metrics to assess the overall performance of competitors, namely, mean fitness ($Mean_{Fit}$), mean accuracy ($Mean_{Acc}$), mean number of selected features ($Mean_{Feat}$), and mean computational time ($Mean_{Time}$) defined as

10 $$\begin{aligned} Mean_{Fit}= & {} \frac{1}{M}\sum _{k=1}^{M} Fit_*^{k}, \end{aligned}$$

11 $$\begin{aligned} Mean_{Acc}= & {} \frac{1}{M} \sum _{k=1}^{M} Acc_*^{k}, \end{aligned}$$

12 $$\begin{aligned} Mean_{Feat}= & {} \frac{1}{M}\sum _{k=1}^{M} \frac{\vert S_*^{k}\vert }{D}, \end{aligned}$$

13 $$\begin{aligned} Mean_{Time}= & {} \frac{1}{M}\sum _{k=1}^{M} Time_*^{k}, \end{aligned}$$

where $M=30$ is the maximum number of independent runs. $f_*^{k}$, $Acc_*^{k}$, $\vert S_*^{k}\vert$, and $Time_*^{k}$ respectively, denote the values of fitness, accuracy, selected feature size, and computational time (measured in milliseconds) for the globally best solution obtained at run *k*.

The smaller the values of $Mean_{Fit}$, $Mean_{Feat}$, and $Mean_{Time}$, the better the CSSA’s performance. In contrast, the higher the value of $Mean_{Acc}$, the greater the CSSA’s performance. The optimality of the results is validated by using the hold-out strategy, in which the training and test sets are realized by randomly dividing each dataset into two parts, with the training phase taking up 80% of the dataset and the testing phase taking up the remaining 20%^73^. Due to the stochastic nature of meta-heuristic algorithms, they cannot be fully replicated, and the average results for each algorithm and single dataset are thus determined over 30 independent runs and realized as the final values for all metrics. Furthermore, we use W, T, and L to represent, respectively, the number of wins, ties, and losses for CSSA in comparison to its rivals across all datasets experimented. Although this may adequately measure the effectiveness of the proposed method, non-parametric statistical tests, such as Wilcoxon’s signed-rank test, Friedman’s rank test, and Nemenyi’s test, are also required to determine CSSA’s statistical significance over its rivals. They are more appropriate and safer than parametric tests since they assume some, if limited, comparability and do not require normal distributions or homogeneity of variance^74^. The best overall performances are indicated in **bold**.

### Comparative analysis

In this section, the $Mean_{Fit}$ of CSSA is compared and examined against the ten various chaotic maps listed in Table 1, in order to obtain the finest CSSA version ever. The $Mean_{Fit}$, $Mean_{Acc}$, $Mean_{Feat}$, and $Mean_{Time}$ are then calculated, and post-hoc statistical analysis is performed on the eighteen UCI datasets and three high-dimensional microarray datasets detailed in Tables 2 and 21, respectively, to see if CSSA has a competitive advantage over its well-known peers. CSSA is also compared to several state-of-the-art, relevant FS methods in the literature to put the acquired results into context. Furthermore, an ablation study is used to do convergence analysis and exploration-exploitation trade-off analysis. The experimental setting has an impact on the final results, and Table 3 summarizes the circumstances for all experiments. There are frequently multiple hyper-parameters in meta-heuristic algorithms, and their values highly affect the performance of the final results to some extent. In this work, all competitors’ algorithm-specific parameter settings match those recommended in their respective papers, with no parameter tuning^75^. Table 4 only provides the parameters that are shared by all algorithms.

**Table 3:** General experimental settings.

Parameter	Value
Operating system	Microsoft Windows 11
Central processing unit (CPU)	11th Gen Intel Core i5-1155G7 2.50 GHz
Random-access memory (RAM)	16GB
Software	Python 3.9.12^62^

**Table 4:** Common parameters for all experiments.

Parameter	Value
Number of independent runs *M*	30
Maximum number of iterations *T*	100
Swarm size *N*	10
Weighting factor $\gamma$ in Eq. (9)	0.99

#### CSSA under different chaotic maps

In this section, the effectiveness of CSSA is investigated under different chaotic maps reported in Table 1 with an initial point ${\tilde{x}}^{0}=0.7$ for all chaotic maps to obey fluctuation patterns^43,69^ and exceptionally ${\tilde{x}}^{0}=0.6$ for the Tent map subjecting to its judgment condition. Thus, the best version of CSSA can be released. *K* in Eq. (5) takes a random value in the range $[-1,1]$ and only the Chebyshev and Iterative maps can, among the ten chaotic maps, give a value in such a range. So, CSSA is separately experimented and results are recorded for the Chebyshev map instead of *K* and Iterative map instead of *K* in Tables 5 and 6, respectively. Since the other three improvements, i.e., generating the initial swarm, substituting for $\alpha$ in Eq. (3), and relocating transgressive sparrows, can be all amended by using random values in the range [0, 1], they can be clearly tested with the ten chaotic maps, conditioned that the Chebyshev and Iterative maps take absolute values. The $Mean_{Fit}$ in Eq. (10) is taken as a key metric in this experiment to measure the distinction between different versions of CSSA based on the ten chaotic maps. We further employ W*, T*, and L* to reflect the advantages and disadvantages of the CSSA’s twenty variants when comparing independently to SSA.

From Tables 5 and 6*combined*, when using the Sinusoidal map, for instance, to substitute for $\alpha$, the experimental results show that CSSA with the Chebyshev and Iterative maps replacing *K* does not perform effectively, with better results than SSA on only 5 and 4 datasets, respectively, indicating that the Sinusoidal map cannot improve SSA’s performance. Furthermore, “W|T|L” shows that the Sinusoidal map has neither wins nor ties on the eighteen datasets when compared to other maps. The experimental results of CSSA under other maps are relatively better than SSA on most datasets. Overall, the best results are obtained when CSSA performs better than SSA on a total of 17 datasets, as shown in Table 5. Thus, since we attempt to maximize the performance of SSA, this study takes the Chebyshev map instead of *K* and the Circle map for the other three improvements, in order to release the best CSSA variant ever based on chaotic maps.

**Table 5:** SSA versus CSSA under different chaotic maps in terms of $Mean_{Fit}$, where the Chebyshev map substitutes for *K* in SSA.

Datasets	Gauss	Circle	Singer	Sinusoidal	Sine	Iterative	Logistic	Piecewise	Chebyshev	Tent	SSA
Breast cancer	0.0206	0.0203	0.0206	0.0206	0.0207	**0.0203**	0.0204	0.0205	0.0206	**0.0203**	0.0208
BreastEW	0.0380	0.0367	0.0368	0.0376	**0.0365**	0.0369	0.0366	0.0366	0.0367	0.0371	0.0371
CongressEW	0.0259	0.0265	0.0261	0.0263	0.0256	0.0261	0.0260	0.0259	0.0273	0.0249	0.0286
Exactly	**0.0046**	0.0060	0.0078	0.0197	**0.0046**	0.0105	0.0063	0.0065	0.0074	0.0070	0.0139
Exactly2	0.2298	0.2211	0.2243	0.2261	0.2231	0.2234	0.2214	0.2211	**0.2203**	0.2226	0.2264
HeartEW	0.0861	0.0860	0.0890	0.0891	0.0884	0.0873	0.0885	0.0901	0.0891	**0.0838**	0.0873
IonosphereEW	0.0682	0.0711	0.0732	0.0798	0.0653	0.0694	0.0702	0.0714	0.0692	0.0781	0.0739
Lymphography	0.1690	**0.1658**	0.1715	0.1786	0.1703	0.1725	0.1679	0.1706	0.1716	0.1714	0.1750
WineEW	0.0032	0.0032	**0.0031**	0.0034	0.0032	**0.0031**	0.0032	0.0032	0.0032	**0.0031**	**0.0031**
Zoo	0.0034	0.0033	0.0033	0.0034	0.0032	0.0033	0.0032	0.0032	0.0033	0.0032	0.0035
M-of-n	**0.0046**	0.0048	0.0047	0.0063	0.0057	0.0047	0.0050	**0.0046**	0.0049	**0.0046**	0.0052
PenglungEW	0.3713	0.3627	0.3779	0.3761	0.3690	0.3737	0.3821	0.3757	0.3714	**0.3517**	0.3715
SonarEW	**0.0164**	0.0188	0.0190	0.0255	0.0186	0.0198	0.0180	0.0205	0.0198	0.0190	0.0225
SpectEW	0.1134	0.1087	0.1141	0.1248	0.1080	0.1125	0.1091	0.1153	0.1119	0.1127	0.1132
Tic-tac-toe	**0.1544**	0.1552	0.1546	0.1552	0.1546	0.1549	0.1552	0.1555	0.1552	0.1552	0.1563
Vote	**0.0022**	0.0024	0.0024	0.0030	0.0027	0.0025	0.0023	0.0024	0.0025	0.0023	0.0025
KrVsKpEW	0.0249	0.0256	0.0260	0.0289	0.0248	0.0251	0.0251	0.0252	0.0265	0.0252	0.0279
WaveformEW	**0.1543**	0.1574	0.1574	0.1629	0.1567	0.1573	0.1581	0.1559	0.1591	0.1574	0.1582
Overall	0.0828	0.0820	0.0840	0.0871	0.0823	0.0835	0.0833	0.0836	0.0833	0.0822	0.0848
W|T|L	**2|4|12**	1|0|17	0|1|17	0|0|18	0|2|16	0|2|16	0|0|18	0|1|17	1|0|17	2|3|13	0|1|17
W*|T*|L*	14|0|4	**17|0|1**	14|1|3	5|0|13	14|0|4	14|3|1	15|0|3	14|0|4	14|1|3	15|2|1	–

Significant values are in [bold].

**Table 6:** SSA vs. CSSA under different chaotic maps in terms of $Mean_{Fit}$, where the Iterative map substitutes for *K* in SSA.

Datasets	Gauss	Circle	Singer	Sinusoidal	Sine	Iterative	Logistic	Piecewise	Chebyshev	Tent	SSA
BreastCancer	0.0205	0.0205	0.0206	0.0206	0.0207	0.0204	0.0206	0.0205	0.0208	**0.0203**	0.0208
BreastEW	0.0379	0.0367	0.0369	0.0376	**0.0365**	0.0372	0.0366	**0.0365**	0.0367	0.0369	0.0371
CongressEW	0.0262	0.0264	0.0253	0.0263	0.0265	0.0251	**0.0247**	0.0261	0.0278	0.0254	0.0286
Exactly	0.0055	**0.0046**	0.0104	0.0248	**0.0046**	0.0083	0.0053	0.0056	0.0067	0.0063	0.0139
Exactly2	0.2275	0.2220	0.2221	0.2267	0.2231	0.2230	0.2212	0.2212	0.2220	0.2221	0.2264
HeartEW	0.0850	0.0877	0.0895	0.089	0.0889	0.0868	0.0895	0.0868	0.0896	0.0850	0.0873
IonosphereEW	**0.0652**	0.0729	0.0725	0.0792	0.0656	0.0674	0.0691	0.0723	0.0706	0.0772	0.0739
Lymphography	0.1722	0.1671	0.1693	0.1839	0.1681	0.1704	0.1746	0.1716	0.1727	0.1704	0.1750
WineEW	**0.0031**	0.0032	0.0032	0.0033	0.0032	0.0032	0.0032	0.0032	0.0033	**0.0031**	**0.0031**
Zoo	0.0034	0.0033	0.0033	0.0034	0.0032	0.0034	**0.0031**	0.0033	0.0033	0.0033	0.0035
M-of-n	**0.0046**	0.0048	0.0047	0.0075	**0.0046**	**0.0046**	**0.0046**	0.0059	0.0046	**0.0046**	0.0052
PenglungEW	0.3667	0.3714	0.3780	0.3760	0.3581	0.3714	0.3712	0.3648	0.3649	0.3714	0.3715
SonarEW	0.0169	0.0189	0.0205	0.0232	0.0187	0.0230	0.0210	0.0204	0.0182	0.0213	0.0225
SpectEW	0.1145	**0.1069**	0.1141	0.1229	0.1086	0.115	0.1098	0.1122	0.1148	0.1164	0.1132
Tic-tac-toe	**0.1544**	0.1552	0.1546	0.1552	0.1546	0.1552	0.1552	0.1546	0.1555	0.1552	0.1563
Vote	**0.0022**	0.0024	0.0023	0.0028	0.0029	0.0024	0.0028	0.0025	0.0026	0.0023	0.0025
KrVsKpEW	**0.0243**	0.0254	0.0273	0.0291	0.0252	0.0261	0.0244	0.0255	0.0256	0.0252	0.0279
WaveformEW	0.1551	0.1570	0.1586	0.163	0.1563	0.1570	0.1561	0.1553	0.1594	0.1572	0.1582
Overall	0.0825	0.0826	0.0841	0.0875	**0.0816**	0.0833	0.0829	0.0827	0.0833	0.0835	0.0848
W|T|L	**2|4|12**	1|1|16	0|0|18	0|0|18	0|3|15	0|1|17	2|1|15	0|1|17	0|0|18	0|3|15	0|1|17
W*|T*|L*	14|1|3	16|0|2	13|0|5	4|0|14	15|0|3	14|0|4	15|0|3	15|1|2	12|1|5	15|1|2	-

Significant values are in [bold].

#### Contribution of chaos to SSA’s overall performance

Table 7 compares the proposed CSSA with SSA based on $Mean_{Fit}$, $Mean_{Acc}$, $Mean_{Feat}$, and $Mean_{Time}$. CSSA gains an outstanding $Mean_{Fit}$ advantage for a total of 17 datasets, and only underperforms SSA on the WineEW dataset. In terms of $Mean_{Acc}$, CSSA obtains the highest accuracy on 14 datasets and similarly for the other 4 ones. In terms of $Mean_{Feat}$, CSSA also outperforms SSA on most datasets. As for $Mean_{Time}$, CSSA relatively has less computational time over the majority of datasets. On the one hand, this implies that the chosen fitness function is able to integrate the role of accuracy and selected feature size in classification tasks. Furthermore, it shows that CSSA can balance the exploration and exploitation capabilities, shielding SSA from falling into local optimum.

**Table 7:** Comparison of CSSA and SSA.

	$Mean_{Fit}$		$Mean_{Acc}$		$Mean_{Feat}$		$Mean_{Time}$	
Dataset	CSSA	SSA	CSSA	SSA	CSSA	SSA	CSSA	SSA
BreastCancer	**0.0204**	0.0208	**0.9857**	0.9855	**0.6300**	0.6400	3420	**3316**
BreastEW	**0.0367**	0.0371	**0.9649**	**0.9649**	**0.1922**	0.2367	**3723**	3934
CongressEW	**0.0265**	0.0286	**0.9762**	0.9739	0.2938	**0.2833**	**2379**	2410
Exactly	**0.0060**	0.0139	**0.9987**	0.9908	**0.4641**	0.4846	5098	**5036**
Exactly2	**0.2211**	0.2264	**0.7815**	0.7768	**0.4795**	0.5487	**5173**	5182
HeartEW	**0.0860**	0.0873	**0.9179**	0.9167	**0.4769**	0.4795	1673	**1653**
IonosphereEW	**0.0711**	0.0739	**0.9315**	0.9286	**0.3245**	0.3275	**3298**	3469
Lymphography	**0.1658**	0.1750	**0.8367**	0.8278	**0.4130**	0.4537	1212	**1197**
WineEW	0.0032	**0.0031**	**1.0000**	**1.0000**	0.3154	**0.3103**	1322	**1303**
Zoo	**0.0033**	0.0035	**1.0000**	**1.0000**	**0.3250**	0.3500	1021	**1006**
M-of-n	**0.0048**	0.0052	**0.9998**	0.9995	**0.4667**	0.4718	5015	**4955**
PenglungEW	**0.3627**	0.3715	**0.6378**	0.6289	0.4098	**0.4089**	**2196**	2239
SonarEW	**0.0188**	0.0225	**0.9849**	0.9817	**0.3906**	0.4400	3082	**3065**
SpectEW	**0.1087**	0.1132	**0.8938**	0.8895	**0.3636**	0.3833	**1860**	1902
Tic-tac-toe	**0.1552**	0.1563	**0.8531**	0.8517	0.9778	**0.9481**	4572	**4357**
Vote	**0.0024**	0.0025	**1.0000**	**1.0000**	**0.2396**	0.2542	**1773**	1780
KrVsKpEW	**0.0256**	0.0279	**0.9800**	0.9780	**0.5750**	0.6120	37344	**36860**
WaveformEW	**0.1574**	0.1582	**0.8468**	0.8463	**0.5800**	0.6042	**37761**	40405
Overall	**0.0820**	0.0848	**0.9216**	0.9189	**0.4399**	0.4576	**6773**	6893
W|T|L	**17|0|1**	1|0|17	**14|4|0**	0|4|14	**14|0|4**	4|0|14	8|0|10	**10|0|8**

Significant values are in [bold].

#### Comparison of CSSA and its peers

This section compares CSSA with twelve well-known algorithms, including SSA, ABC, PSO, BA, WOA, GOA, HHO, BSA, ASO, HGSO, LSHADE, and CMAES, in order to determine whether CSSA has a competitive advantage over them. A brief description of compared algorithms is given in Table 8.

**Table 8:** Summary information about the twelve compared optimization algorithms.

Algorithm	Acronym	Inspiration	Year
Particle swarm optimization^21^	PSO	Intelligent, collective, social behavior of bird and fish flocks	1995
Evolution strategy with covariance matrix adaptation^59^	CMAES	Adaptively adjusting the covariance matrix	2003
Artificial bee colony^23^	ABC	Foraging behavior of bees	2005
Bat algorithm^28^	BA	Behavior of bats during foraging	2010
Success-history based adaptive differential evolution with linear population size reduction^58^	LSHADE	An improved variant of the differential evolution algorithm	2014
Whale optimization algorithm^24^	WOA	The social behavior of humpback whales	2016
Bird swarm algorithm^27^	BSA	Strategies of bird flocks during foraging and migration	2016
Grasshopper optimization algorithm^25^	GOA	Grasshopper strategies for foraging and mating	2017
Atom search optimization^29^	ASO	Motion behavior of atoms in the search space	2019
Harris hawks optimization^26^	HHO	Cooperative behavior and chasing style of Harris’ hawks	2019
Henry gas solubility optimization^30^	HGSO	The behavior governed by Henry’s law	2019
Sparrow search algorithm^45^	SSA	Foraging and anti-predatory behaviors of sparrows	2020

Table 9 compares the $Mean_{Fit}$ of CSSA with that of its peers. The results show that CSSA obtains the smallest $Mean_{Fit}$ on 13 datasets and ABC, SSA, and CMAES perform relatively better on the remaining datasets. Thus, $Mean_{Fit}$ results show that CSSA holds its own merits for most datasets and can perform best in comparison to other rivals by adapting itself to classification tasks.

**Table 9:** Comparison of CSSA against its peers in terms of $Mean_{Fit}$.

Dataset	CSSA	SSA	ABC	PSO	BA	WOA	GOA	HHO	BSA	ASO	HGSO	LSHADE	CMAES
BreastCancer	0.0204	0.0208	**0.0202**	0.0227	0.0238	0.0208	0.0216	0.0217	0.0213	0.0300	0.0248	0.0213	0.0205
BreastEW	**0.0367**	0.0371	0.0380	0.0395	0.0417	0.0380	0.0388	0.0389	0.0382	0.0490	0.0467	0.0388	0.0415
CongressEW	0.0265	0.0286	**0.0260**	0.0317	0.0350	0.0278	0.0291	0.0297	0.0276	0.0383	0.0370	0.0303	0.0295
Exactly	**0.0060**	0.0139	0.0209	0.1213	0.1695	0.0272	0.0547	0.0399	0.0588	0.2628	0.1651	0.0570	0.0331
Exactly2	0.2211	0.2264	**0.2160**	0.2276	0.2385	0.2207	0.2251	0.2316	0.2223	0.2433	0.2413	0.2300	0.2280
HeartEW	**0.0860**	0.0873	0.0864	0.1047	0.1154	0.0903	0.0904	0.0924	0.0912	0.1439	0.1142	0.0923	0.0896
IonosphereEW	**0.0711**	0.0739	0.0815	0.0973	0.1070	0.0840	0.0902	0.0812	0.0893	0.1116	0.1125	0.0879	0.0957
Lymphography	**0.1658**	0.1750	0.1674	0.1964	0.2128	0.1848	0.1775	0.1857	0.1861	0.2297	0.2118	0.1881	0.1833
WineEW	0.0032	**0.0031**	0.0034	0.0052	0.0141	0.0036	0.0036	0.0035	0.0036	0.0322	0.0112	0.0035	0.0039
Zoo	**0.0033**	0.0035	0.0035	0.0042	0.0126	0.0037	0.0038	0.0038	0.0038	0.0189	0.0054	0.0038	0.0041
M-of-n	**0.0048**	0.0052	0.0058	0.0480	0.0525	0.0097	0.0158	0.0129	0.0150	0.1397	0.0597	0.0149	0.0099
PenglungEW	**0.3627**	0.3715	0.3720	0.3983	0.4027	0.3916	0.3895	0.3845	0.3873	0.4024	0.4012	0.3982	0.4017
SonarEW	**0.0188**	0.0225	0.0251	0.0438	0.0533	0.0278	0.0313	0.0261	0.0305	0.0629	0.0495	0.0322	0.0282
SpectEW	**0.1087**	0.1132	0.1137	0.1367	0.1516	0.1208	0.1265	0.1297	0.1304	0.1652	0.1527	0.1252	0.1265
Tic-tac-toe	0.1552	0.1563	**0.1544**	0.1587	0.1671	0.1565	0.1546	0.1592	0.1552	0.1854	0.1584	0.1552	**0.1544**
Vote	**0.0024**	0.0025	0.0038	0.0110	0.0169	0.0030	0.0033	0.0047	0.0049	0.0218	0.0181	0.0053	0.0059
KrVsKpEW	**0.0256**	0.0279	0.0278	0.0386	0.0410	0.0292	0.0323	0.0286	0.0323	0.0708	0.0400	0.0340	0.0281
WaveformEW	**0.1574**	0.1582	0.1628	0.1787	0.1788	0.1627	0.1685	0.1634	0.1696	0.1902	0.1788	0.1691	0.1632
Overall	**0.0820**	0.0848	0.0849	0.1036	0.1130	0.0890	0.0920	0.0910	0.0926	0.1332	0.1127	0.0937	0.0915
W|T|L	**13|0|5**	1|0|17	3|1|14	0|0|18	0|0|18	0|0|18	0|0|18	0|0|18	0|0 |18	0|0|18	0|0|18	0|0|18	0|1|17

Significant values are in [bold].

Table 10 compares CSSA with other algorithms in terms of $Mean_{Acc}$. The comparison results illustrate that CSSA obtains the highest $Mean_{Acc}$ on 9 datasets, ties for the highest on 6 datasets, having thus an outstanding performance on a total of 15 datasets, while ABC solely have higher $Mean_{Acc}$ than CSSA on only 3 datasets: CongressEW, Exactly2, and Tic-tac-toe. On the other hand CMAES only performs better than CSSA on the Tic-tac-toe. This may be attributed to the complex nature of data in these datasets.

**Table 10:** Comparison of CSSA against its peers in terms of $Mean_{Acc}$.

Dataset	CSSA	SSA	ABC	PSO	BA	WOA	GOA	HHO	BSA	ASO	HGSO	LSHADE	CMAES
BreastCancer	**0.9857**	0.9855	**0.9857**	0.9838	0.9826	**0.9857**	0.9850	0.9855	0.9850	0.9757	0.9824	0.9852	**0.9857**
BreastEW	**0.9649**	**0.9649**	**0.9649**	0.9640	0.9620	0.9646	0.9643	0.9635	**0.9649**	0.9550	0.9579	0.9646	0.9629
CongressEW	0.9762	0.9739	**0.9774**	0.9716	0.9678	0.9759	0.9739	0.9728	0.9755	0.9648	0.9667	0.9724	0.9743
Exactly	**0.9987**	0.9908	0.9840	0.8837	0.8350	0.9777	0.9503	0.9650	0.9462	0.7413	0.8398	0.9724	0.9718
Exactly2	0.7815	0.7768	**0.7870**	0.7757	0.7640	0.7822	0.7775	0.7713	0.7808	0.7585	0.7617	0.7727	0.7757
HeartEW	**0.9179**	0.9167	**0.9179**	0.8988	0.8883	0.9136	0.9136	0.9117	0.9130	0.8593	0.8901	0.9117	0.9148
IonosphereEW	**0.9315**	0.9286	0.9216	0.9061	0.8962	0.9188	0.9127	0.9216	0.9136	0.8911	0.8911	0.9150	0.9080
Lymphography	**0.8367**	0.8278	0.8356	0.8067	0.7900	0.8178	0.8256	0.8167	0.8167	0.7733	0.7922	0.8144	0.8200
WineEW	**1.0000**	**1.0000**	**1.0000**	0.9991	0.9898	**1.0000**	**1.0000**	**1.0000**	**1.0000**	0.9722	0.9944	**1.0000**	**1.0000**
Zoo	**1.0000**	**1.0000**	**1.0000**	**1.0000**	0.9921	**1.0000**	**1.0000**	**1.0000**	**1.0000**	0.9857	**1.0000**	**1.0000**	**1.0000**
M-of-n	**0.9998**	0.9995	0.9992	0.9578	0.9532	0.9953	0.9895	0.9922	0.9903	0.8657	0.9468	0.9903	0.9953
PenglungEW	**0.6378**	0.6289	0.6289	0.6022	0.5978	0.6089	0.6111	0.6156	0.6133	0.5978	0.6000	0.6022	0.6000
SonarEW	**0.9849**	0.9817	0.9794	0.9603	0.9508	0.9762	0.9730	0.9778	0.9738	0.9413	0.9556	0.9722	0.9770
SpectEW	**0.8938**	0.8895	0.8895	0.8660	0.8512	0.8821	0.8765	0.8728	0.8722	0.8377	0.8512	0.8778	0.8772
Tic-tac-toe	0.8531	0.8517	**0.8542**	0.8486	0.8391	0.8514	0.8538	0.8479	0.8531	0.8194	0.8493	0.8531	**0.8542**
Vote	**1.0000**	**1.0000**	0.9994	0.9922	0.9861	**1.0000**	**1.0000**	0.9983	0.9983	0.9811	0.9861	0.9978	0.9978
KrVsKpEW	**0.9800**	0.9780	0.9783	0.9670	0.9645	0.9764	0.9735	0.9773	0.9735	0.9341	0.9667	0.9716	0.9783
WaveformEW	**0.8468**	0.8463	0.8418	0.8250	0.8254	0.8415	0.8356	0.8411	0.8345	0.8142	0.8258	0.8352	0.8416
Overall	**0.9216**	0.9189	0.9192	0.9005	0.8909	0.9149	0.9120	0.9128	0.9114	0.8705	0.8921	0.9102	0.9130
W|T|L	**9|6|3**	0|4|14	3|5|10	0|1|17	0|0|18	0|4|14	0|3|15	0|2|16	0|3|15	0|0|18	0|1|17	0|2|16	0|4|14

Significant values are in [bold].

Table 11 compares CSSA with its peers in terms of $Mean_{Feat}$. CSSA has the lowest number of features selected on 9 datasets, while the other 12 algorithms won only on 9 datasets. Noteworthily, ABC is second to CSSA in terms of only $Mean_{Fit}$ and $Mean_{Acc}$, but has no advantages in terms of $Mean_{Feat}$.

**Table 11:** Comparison of CSSA against its peers in terms of $Mean_{Feat}$.

Dataset	CSSA	SSA	ABC	PSO	BA	WOA	GOA	HHO	BSA	ASO	HGSO	LSHADE	CMAES
BreastCancer	0.6300	0.6400	0.6067	0.6667	0.6633	0.6633	0.6733	0.7300	0.6467	**0.6000**	0.7367	0.6700	0.6333
BreastEW	**0.1922**	0.2367	0.3289	0.3889	0.4078	0.2967	0.3478	0.2711	0.3500	0.4467	0.5000	0.3733	0.4733
CongressEW	0.2938	0.2833	0.3604	0.3583	0.3188	0.3854	0.3292	**0.2792**	0.3333	0.3417	0.4042	0.3021	0.4062
Exactly	**0.4641**	0.4846	0.5077	0.6103	0.6128	0.5051	0.5564	0.5231	0.5487	0.6744	0.6538	0.5538	0.5231
Exactly2	0.4795	0.5487	0.5179	0.5462	0.4821	0.5000	0.4821	0.5179	0.5308	**0.4179**	0.5385	0.4897	0.5923
HeartEW	0.4769	0.4795	0.5077	**0.4513**	0.4821	0.4769	0.4872	0.5000	0.5077	0.4564	0.5410	0.4923	0.5282
IonosphereEW	**0.3245**	0.3275	0.3833	0.4294	0.4324	0.3588	0.3745	0.3588	0.3765	0.3765	0.4706	0.3784	0.4618
Lymphography	**0.4130**	0.4537	0.4574	0.4963	0.4870	0.4444	0.4759	0.4241	0.4630	0.5333	0.6093	0.4444	0.5056
WineEW	0.3154	**0.3103**	0.3385	0.4308	0.3974	0.3564	0.3590	0.3462	0.3615	0.4692	0.5667	0.3538	0.3949
Zoo	**0.3250**	0.3500	0.3479	0.4167	0.4708	0.3667	0.3792	0.3771	0.3750	0.4792	0.5438	0.3771	0.4062
M-of-n	**0.4667**	0.4718	0.4949	0.6282	0.6154	0.5051	0.5359	0.5154	0.5462	0.6718	0.7077	0.5308	0.5231
PenglungEW	0.4098	0.4089	0.4624	0.4482	0.4492	0.4389	0.4452	**0.3904**	0.4466	0.4203	0.5224	0.4414	0.5725
SonarEW	**0.3906**	0.4400	0.4656	0.4483	0.4550	0.4278	0.4617	0.4100	0.4594	0.4800	0.5500	0.4722	0.5389
SpectEW	**0.3636**	0.3833	0.4273	0.4045	0.4318	0.4045	0.4258	0.3788	0.3909	0.4515	0.5409	0.4152	0.4924
Tic-tac-toe	0.9778	0.9481	1.0000	0.8815	0.7741	0.9407	0.9926	0.8667	0.9778	**0.6667**	0.9185	0.9778	1.0000
Vote	**0.2396**	0.2542	0.3208	0.3271	0.3104	0.3042	0.3250	0.3062	0.3208	0.3125	0.4375	0.3125	0.3688
KrVsKpEW	0.5750	0.6120	0.6269	0.5981	0.5917	0.5843	0.6074	0.6074	0.6111	**0.5509**	0.6963	0.5861	0.6630
WaveformEW	0.5800	0.6042	0.6225	**0.5492**	0.5867	0.5817	0.5775	0.6083	0.5683	0.6267	0.6342	0.5958	0.6342
Overall	**0.4399**	0.4576	0.4876	0.5044	0.4983	0.4745	0.4909	0.4673	0.4897	0.4987	0.5873	0.4870	0.5399
W|T|L	**9|0|9**	1|0|17	0|0|18	2|0|16	0|0|18	0|0|18	0|0|18	2|0|16	0|0|18	4|0|14	0|0|18	0|0|18	0|0|18

Significant values are in [bold].

Table 12 compares $Mean_{Time}$ of CSSA over other algorithms. LSHADE has the lowest $Mean_{Time}$ among all algorithms, but the algorithm performs poorly in other aspects such as $Mean_{Fit}$, $Mean_{Acc}$, and $Mean_{Feat}$. While ABC performs slightly better for these metrics, it has the longest run time, reaching almost three times the duration of CSSA. In addition, although the $Mean_{Time}$ of CSSA is in the middle of the range of all the algorithms compared, it has a lower time cost than standard SSA, as shown in Table 7). This shows that CSSA significantly improves the performance of SSA without increasing or even decreasing the time complexity of the algorithm. This is another aspect that demonstrates the advantage of CSSA over standard one.

**Table 12:** Results of CSSA compared to its peers in terms of $Mean_{Time}$.

Dataset	CSSA	SSA	ABC	PSO	BA	WOA	GOA	HHO	BSA	ASO	HGSO	LSHADE	CMAES
BreastCancer	3420	3316	9351	2958	2337	3249	3431	2646	2973	2834	3322	**1977**	3042
BreastEW	3723	3934	11900	3729	2975	3843	4805	2688	3804	3843	4239	**2439**	5960
CongressEW	2379	2410	6822	2253	1783	2362	2795	1806	2190	2285	2480	**1388**	2252
Exactly	5098	5036	14660	4448	3617	5029	5181	4003	4598	4179	5165	**2859**	4927
Exactly2	5173	5182	15151	4732	3811	5094	5128	3860	4562	4798	5258	**2893**	4938
HeartEW	1673	1653	4621	1528	1216	1625	2013	1280	1501	1505	1672	**996**	1557
IonosphereEW	3298	3469	10371	3362	2652	3465	4330	2509	3260	3485	3825	**2051**	5507
Lymphography	1212	1197	3305	1110	873	1164	1717	934	1083	1080	1210	**735**	1143
WineEW	1322	1303	3616	1207	958	1281	1693	1016	1185	1181	1323	**797**	1235
Zoo	1021	1006	2776	940	740	984	1495	791	918	913	1021	**633**	970
M-of-n	5015	4955	14514	4378	3474	4984	5088	4036	4539	4069	5124	**2927**	5001
PenglungEW	2196	2239	6264	2220	1667	2200	11317	1746	2036	2073	2259	**1308**	5554
SonarEW	3082	3065	8544	2828	2232	3008	4505	2353	2784	2812	3109	**1812**	4333
SpectEW	1860	1902	5357	1767	1334	1846	2522	1391	1707	1641	1915	**1307**	1951
Tic-tac-toe	4572	4357	13120	3965	3118	4390	4532	3584	4068	3605	4597	**2600**	4385
Vote	1773	1780	5021	1663	1312	1738	2206	1360	1622	1673	1832	**1102**	1704
KrVsKpEW	37344	36860	105984	32993	25971	36520	35067	28855	33384	29439	37637	**23292**	34776
WaveformEW	37761	40405	99308	38708	33715	37703	37206	28685	35288	56568	37046	**22749**	33595
Overall	6773	6893	18927	6377	5210	6694	7502	5197	6195	7110	6835	**4104**	6824
W|T|L	0|0|18	0|0|18	0|0|18	0|0|18	0|0|18	0|0|18	0|0|18	0|0|18	0|0|18	0|0|18	0|0|18	**18|0|0**	0|0|18

Significant values are in [bold].

**Figure 3:**
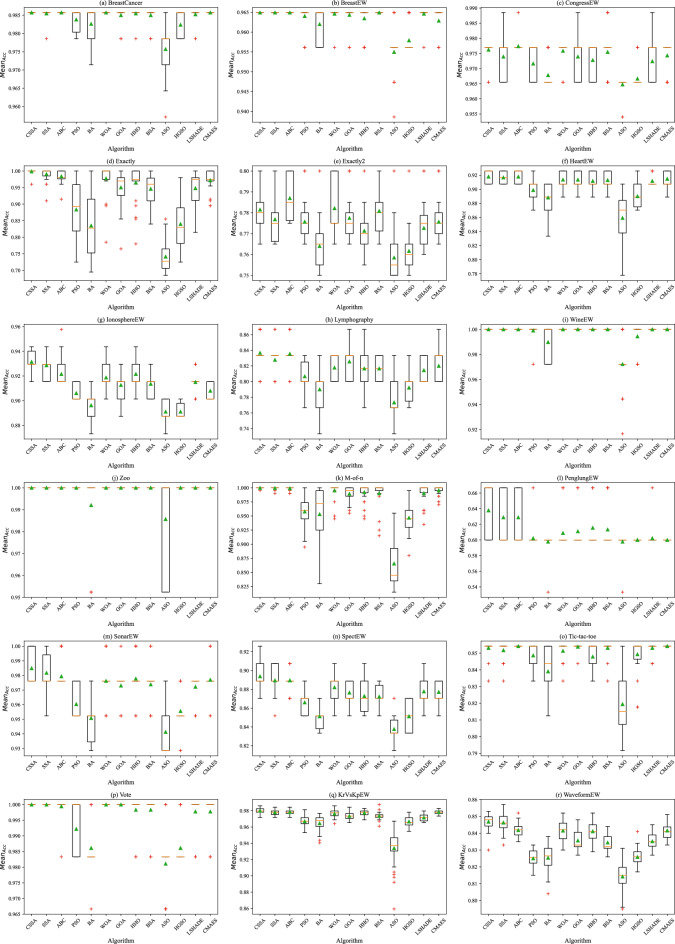
Boxplot of $$Mean_{Acc}$$.

**Figure 4:**
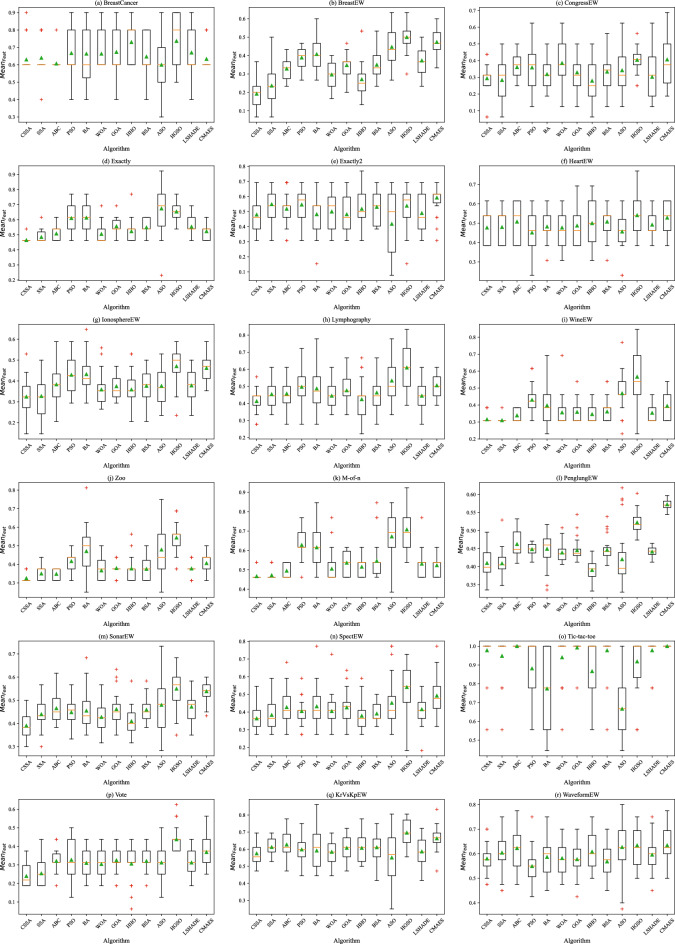
Boxplot of $$Mean_{Feat}$$.

**Figure 5:**
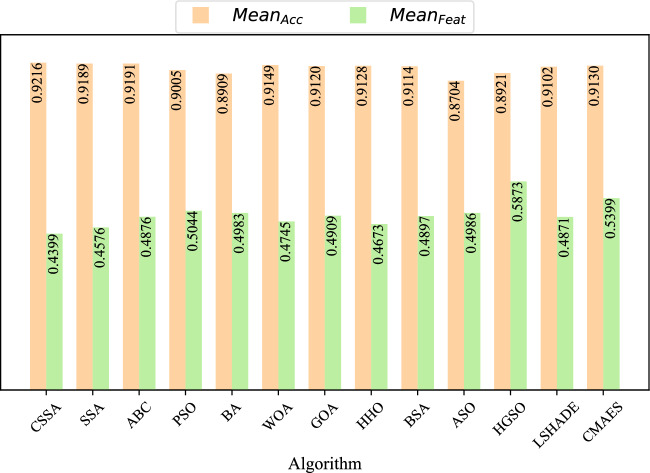
Bar chart of $Mean_{Acc}$ and $Mean_{Feat}$.

Furthermore, Figs. 3 and 4 prove the stability of CSSA in terms of $Mean_{Acc}$ and $Mean_{Feat}$ in means of boxplots. As can be seen from Fig. 3, CSSA obtained higher boxplots on all datasets except Exactly2. On the other hand, CSSA has smaller box sizes on all datasets except PenglungEW, SonarEW, and SpectEW, indicating that CSSA is more stable in terms of $Mean_{Acc}$ compared to its peers. Figure 4 also shows that CSSA is able to achieve lower $Mean_{Feat}$ on most datasets, guaranteeing a lower size of the boxplots. Figure 5 shows $Mean_{Acc}$ and $Mean_{Feat}$ of all competitors. It can be seen that CSSA achieves the highest $Mean_{Acc}$ accompanied with the least $Mean_{Feat}$.

#### Convergence curves of all competitors

**Figure 6:**
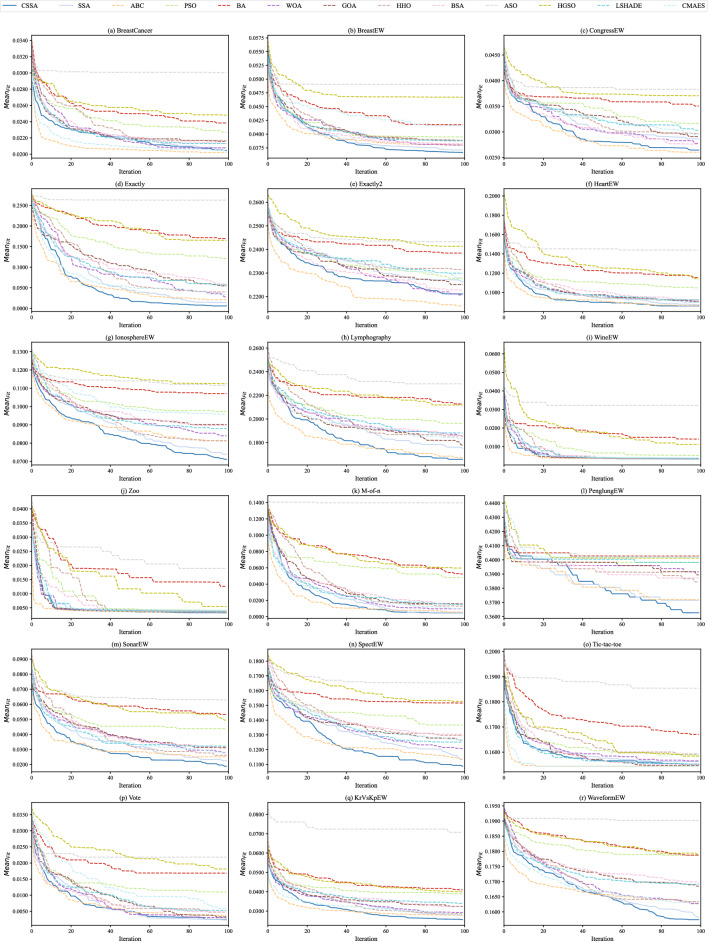
Convergence curves of CSSA and its peers.

Aforementioned experimental results can effectively describe the subtle differences among competing algorithms, but we also need to control the algorithm as a whole. The convergence behavior of all competitors is further analyzed. Figure 6 visually compares the $Mean_{Fit}$ trace of all competitors for the eighteen datasets, where all results are the mean of 30 independent runs per each iteration. It is clear that CSSA is more effective compared to SSA on almost all datasets, exhibiting that the convergence of CSSA is more accelerated than that of its peers. For most datasets, CSSA is at the bottom of the convergence traces of all other eleven algorithms, indicating that CSSA holds a competitive advantage among its rivals in terms of rapid convergence while jumping out of the local optima. This may be due to the distinctive characteristics (especially ergodicity) of chaotic maps, which help cover the whole search space more conveniently. Thus, CSSA achieves better exploratory and exploitative behaviors than its peers.

### Statistical test and analysis

Although it is evident from the previous analysis that CSSA has significant advantages over its peers, further statistical tests of the experimental results are required to bring rigorousness in terms of stability and reliability analyses. In this study, we analyze whether CSSA has a statistically significant advantage over its peers based on a *p*-value by using the Wilcoxon’s signed-rank test at a 5% significance level^76^. When *p*<0.05, this indicates a significant advantage of CSSA compared to its peers; otherwise, CSSA has a comparable effectiveness among all competitors.

Table 13 shows the results of the Wilcoxon’s signed-rank test for CSSA over other competitors in terms of $Mean_{Fit}$, where “+” represents the number of datasets on which CSSA has a significant advantage over its peers, “$\approx$” indicates that CSSA is comparable to the corresponding competing algorithm, and “−” represents the number of datasets on which CSSA works worse than the algorithm it is being compared against. From Table 13, it is clear that CSSA has outstanding advantages over PSO, BA, HHO, and ASO for all the eighteen datasets, and over SSA, HGSO, LSHADE, CMAES, GOA, BSA, WOA, and ABC on 7, 17, 17, 16, 16, 16, 15, 14, and 12 datasets, respectively. Thus, CSSA outperforms its peers significantly on most datasets.

**Table 13:** *p*-values of Wilcoxon’s signed-rank test on CSSA vs. its peers in terms of $Mean_{Fit}$.

Dataset	SSA	ABC	PSO	BA	WOA	GOA	HHO	BSA	ASO	HGSO	LSHADE	CMAES
BreastCancer	3.34E-01	1.57E-01	**3.36E-04**	**4.54E-05**	2.36E-01	**1.41E-03**	**7.25E-04**	**1.16E-02**	**3.57E-06**	**2.32E-06**	**7.60E-03**	1.00E+00
BreastEW	**2.89E-02**	**3.41E-06**	**1.68E-06**	**2.52E-06**	**5.90E-05**	**1.70E-06**	**9.71E-04**	**2.63E-06**	**1.72E-06**	**1.70E-06**	**2.00E-06**	**2.00E-06**
CongressEW	**2.06E-02**	2.64E-01	**4.88E-05**	**3.39E-06**	**5.44E-04**	**7.95E-03**	**1.89E-03**	**4.54E-02**	**1.68E-06**	**2.15E-06**	**9.50E-05**	**7.30E-05**
Exactly	6.30E-02	**2.11E-03**	**2.55E-06**	**2.56E-06**	**1.74E-03**	**4.99E-06**	**1.31E-03**	**1.76E-05**	**1.73E-06**	**1.72E-06**	**1.20E-05**	**4.28E-04**
Exactly2	**2.97E-02**	6.76E-02	**1.79E-02**	**1.23E-05**	9.04E-01	7.09E-02	**4.59E-04**	5.74E-01	**5.88E-06**	**7.98E-06**	**1.27E-03**	**5.45E-03**
HeartEW	2.63E-01	1.92E-01	**2.01E-05**	**1.70E-06**	**2.40E-02**	**1.35E-02**	**3.70E-03**	**4.93E-03**	**1.71E-06**	**3.43E-06**	**3.60E-04**	**1.20E-02**
IonosphereEW	2.10E-01	**2.04E-04**	**2.00E-06**	**1.72E-06**	**4.35E-05**	**3.17E-06**	**2.33E-03**	**1.63E-05**	**1.73E-06**	**1.73E-06**	**2.00E-05**	**2.00E-06**
Lymphography	**1.91E-02**	2.79E-01	**5.53E-06**	**3.77E-06**	**5.51E-05**	**4.24E-03**	**1.14E-03**	**1.04E-05**	**1.71E-06**	**1.72E-06**	**8.00E-05**	**1.60E-04**
WineEW	3.17E-01	**2.01E-02**	**2.66E-05**	**3.91E-05**	**5.10E-03**	**3.16E-03**	**1.06E-02**	**1.83E-03**	**1.65E-06**	**1.62E-06**	**5.49E-03**	**1.51E-04**
Zoo	**2.70E-03**	**4.51E-03**	**5.65E-06**	**3.50E-06**	**1.57E-03**	**4.15E-05**	**6.57E-04**	**9.89E-04**	**3.54E-06**	**1.55E-06**	**9.00E-05**	**1.40E-05**
M-of-n	2.76E-01	**1.35E-02**	**2.52E-06**	**8.11E-06**	**2.07E-02**	**8.82E-05**	**1.17E-03**	**1.27E-04**	**1.73E-06**	**1.72E-06**	**3.33E-04**	**6.33E-04**
PenglungEW	3.44E-01	**7.97E-03**	**1.73E-06**	**2.60E-06**	**2.83E-05**	**1.60E-05**	**1.43E-02**	**1.02E-05**	**2.84E-05**	**1.73E-06**	**2.00E-06**	**2.00E-06**
SonarEW	**4.74E-02**	**2.17E-04**	**1.73E-06**	**2.11E-06**	**2.34E-04**	**1.72E-06**	**1.57E-02**	**3.49E-06**	**1.73E-06**	**1.73E-06**	**2.00E-06**	**4.80E-05**
SpectEW	3.49E-01	**4.62E-02**	**5.28E-06**	**1.72E-06**	**2.55E-03**	**2.83E-05**	**1.38E-04**	**8.78E-06**	**1.72E-06**	**1.72E-06**	**1.41E-04**	**2.30E-05**
Tic-tac-toe	1.80E-01	1.80E-01	**1.03E-02**	**1.74E-04**	2.12E-01	4.14E-01	**1.53E-02**	1.00E-00	**3.56E-06**	8.48E-02	1.00E+00	1.02E-01
Vote	2.98E-01	**7.31E-05**	**2.05E-06**	**3.32E-06**	**7.81E-04**	**2.40E-04**	**1.09E-04**	**2.81E-05**	**1.65E-06**	**1.35E-06**	**4.60E-05**	**3.00E-06**
KrVsKpEW	**4.83E-03**	**2.01E-02**	**2.13E-06**	**1.73E-06**	**1.14E-02**	**3.40E-05**	**3.61E-03**	**9.78E-06**	**1.73E-06**	**1.73E-06**	**3.00E-06**	**5.67E-03**
WaveformEW	5.17E-01	**2.51E-04**	**1.73E-06**	**1.73E-06**	**2.10E-03**	**6.98E-06**	**9.49E-05**	**1.92E-06**	**1.73E-06**	**1.73E-06**	**7.00E-06**	**1.01E-04**
$+$|$\approx$|−	7|11|0	12|6|0	18|0|0	18|0|0	15|3|0	16|2|0	18|0|0	16|2|0	18|0|0	17|1|0	17|1|0	16|2|0

Significant values are in [bold].

In addition, we further measures the statistical significance of CSSA relative to other algorithms in terms of $Mean_{Fit}$ by Friedman’s rank test^77^. Assuming that we take a significance level $\alpha =0.05$, Friedman’s rank test is measured as

14 $$\begin{aligned} \chi _{F}^{2}=\frac{12 N_{D}}{N_{A}(N_{A}+1)}\left( \sum _{k=1}^{N_{A}} R_{k}^{2}-\frac{N_{A}(N_{A}+1)^{2}}{4}\right) , \end{aligned}$$

which is undesirably conservative, and a better statistic is therefore derived as^78^

15 $$\begin{aligned} F_{F}=\frac{(N_{D}-1) \chi _{F}^{2}}{N_{D}(N_{A}-1)-\chi _{F}^{2}}, \end{aligned}$$

where $N_{D}$ is the number of datasets, $N_{A}$ is the number of comparative algorithms, and $R_{k}$ is the average ranking of an algorithm *k*. Thus, we have $N_{D} = 18$, $N_{A} = 13$, and $R_{k}$ calculated from Tables 9, 10, 11, and 12. Table 14 shows $R_{k}$, $\chi _{F}^{2}$, and $F_{F}$ for all algorithms under our four evaluation metrics. $F_{F}$ obeys the *F*-distribution with degrees of freedom $N_{A}-1$ and $(N_{A}-1)(N_{D}-1)$. The calculation gives $F(12,204)=1.80$, and since all $F_{F}$ are greater than that value, there is a significant difference among the algorithms in favor of CSSA.

**Table 14:** Results of Friedman’s rank test on CSSA vs. its peers.

Metric	CSSA	SSA	ABC	PSO	BA	WOA	GOA	HHO	BSA	ASO	HGSO	LSHADE	CMAES	$\chi _{F}^{2}$	$F_{F}$
$Mean_{Fit}$	1.50	2.94	2.64	9.72	11.58	4.72	6.39	6.72	6.69	12.89	11.36	7.33	6.50	182.35	92.11
$Mean_{Acc}$	2.17	3.67	2.83	9.56	11.58	4.86	6.25	6.64	6.50	12.94	10.94	7.42	5.64	163.41	52.83
$Mean_{Feat}$	2.25	4.28	7.39	7.92	7.67	4.81	7.39	4.86	7.19	7.56	12.19	6.81	10.69	97.34	13.94
$Mean_{Time}$	9.17	9.06	12.94	5.89	2.22	7.89	11.11	2.83	4.94	5.44	10.44	1.00	8.06	188.57	116.85

Friedman’s rank test alone is usually unable to compare the significance of the algorithms against each other. So, Nemenyi’s test is also conducted^74^. This test essentially compares the difference between the average ranking of each algorithm with a critical difference *CD*. If the difference is greater than *CD*, it indicates that the algorithm with the lower ranking is superior; otherwise, there is no statistical difference between the algorithms. *CD* is calculated as

16 $$\begin{aligned} CD = q_{\alpha }\sqrt{\frac{N_{A}(N_{A}+1)}{6N_{D}}}, \end{aligned}$$

where $q_{\alpha }$ is calculated as 3.31, given that $N_{A}=13$ and the confidence level $\alpha =0.05$. Thus, $CD = 4.30$, and significant differences between two algorithms hold when the difference between their average ranking is greater than that value.

**Figure 7:**
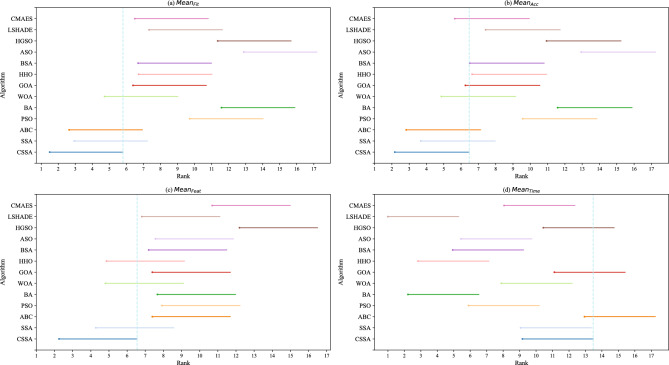
Nemenyi’s test on CSSA against its peers in terms of $Mean_{Fit}$, $Mean_{Acc}$, $Mean_{Feat}$, and $Mean_{Time}$.

Figure 7 shows *CD* results for all competitors. Vertical dots indicate the average ranking of the algorithms, and the horizontal line segment starting with the point indicates the critical difference. A significant difference between the algorithms is represented by the absence of intersection of the horizontal line segments of the algorithms. As shown, CSSA performs best in terms of $Mean_{Fit}$, $Mean_{Acc}$ and $Mean_{Feat}$, but performs less well in terms of $Mean_{Time}$. CSSA intersects only SSA, ABC and WOA in terms of $Mean_{Fit}$ and only SSA, WOA and HHO in terms of $Mean_{Feat}$, indicating that CSSA is significantly different from most compared algorithms in terms of $Mean_{Fit}$ and $Mean_{Feat}$. On the other hand, Fig. 7b shows that CSSA is significantly different from PSO, BA, HHO, ASO, HGSO and LSHADE in terms of $Mean_{Acc}$, and Fig. 7d shows that there is no significant advantage in $Mean_{Time}$ for CSSA, but rather a significant advantage for LSHADE. Furthermore, there is a difference between CSSA and SSA though it is not significant. Overall, since the $Mean_{Fit}$ among all evaluation metrics can synthesize the ability of the algorithm to handle FS problems, Wilcoxon’s signed-rank test, Friedman’s rank test, and Nemenyi’s test show that CSSA has a satisfactorily significant performance over its peers.

### Merits of CSSA’s main components via an ablation study

In this experiment, five representative continuous benchmark functions are picked from the CEC benchmark suite to investigate the impact of the different improvements embedded into CSSA in terms of swarm diversity and convergence trace. Their characteristics and mathematical definitions are reported in Table 15.

**Table 15:** Five representative CEC benchmark functions with diverse characteristics.

Function	Characteristics	Mathematical expression	Limits	$f_{min}$
Ackley	⋅ Uni-modal ⋅ Differentiable ⋅ Convex ⋅ Non-separable	$f(\textbf{x})=-20\exp \left( -0.2\sqrt{\frac{1}{D}\sum _{j=1}^{D}x_{j}^{2}}\right) -\exp {\left( \frac{1}{D}\sum _{j=1}^{D}\cos {(2\pi x_{j})}\right) +20+\exp {(1)}}$	$[-32.768, 32.768]$	0
Rastrigin	⋅ Multi-modal ⋅ Differentiable ⋅ Convex ⋅ Separable	$f({\textbf{x}})=10d+\sum _{j=1}^{D}[x_{j}^{2}-10\cos {(2\pi x_j)}]$	$[-5.12, 5.12]$	0
Rosenbrock	⋅ Multi-modal ⋅ Differentiable ⋅ Non-convex ⋅ Non-separable	$f({\textbf{x}})=\sum _{j=1}^{D-1}[100(x_{j+1}-x_{j}^{2})^{2}+(x_{j}-1)^{2}]$	$[-2.048, 2.048]$	0
Shekel	⋅ Multi-modal	$f({\textbf{x}})=-\sum _{i=1}^{10}\left( B_{i}+\sum _{j=1}^{4}(x_{j}-\textrm{C}_{ji})^{2}\right) ^{-1}$, $B=\frac{1}{10}(1,2,2,4,4,6,3,7,5,5)^T$, $$ {\textbf{C}}=\left( \begin{array}{llllllllll} 4.0 &{} 1.0 &{} 8.0 &{} 6.0 &{} 3.0 &{} 2.0 &{} 5.0 &{} 8.0 &{} 6.0 &{} 7.0 \\ 4.0 &{} 1.0 &{} 8.0 &{} 6.0 &{} 7.0 &{} 9.0 &{} 3.0 &{} 1.0 &{} 2.0 &{} 3.6 \\ 4.0 &{} 1.0 &{} 8.0 &{} 6.0 &{} 3.0 &{} 2.0 &{} 5.0 &{} 8.0 &{} 6.0 &{} 7.0 \\ 4.0 &{} 1.0 &{} 8.0 &{} 6.0 &{} 7.0 &{} 9.0 &{} 3.0 &{} 1.0 &{} 2.0 &{} 3.6 \end{array}\right) $$	[0,10]	−10.5364
Himmelblau	⋅ Multi-modal ⋅ Non-convex	$f({\textbf{x}})=(x_{1}^{2}+x_{2}-11)^{2}+(x_{1}+x_{2}^{2}-7)^{2}$	$[-5, 5]$	0

Since CSSA is specifically proposed for FS problems, its search space is restricted to [0, 1] due to the existence of chaotic maps. However, in order to fully demonstrate the advantages of its main components, CSSA should be tested in different search spaces for diverse benchmark functions. Therefore, we further analyze CSSA in comparison to CSSA without chaotic initial swarm (NINICSSA), CSSA without chaotic random parameters (NPARCSSA), and CSSA without chaotic update of transgressive positions (NPOSCSSA). We define parameter settings in this experiment for all algorithms as: the maximum number of iterations is 100, swarm size is 30, and $D=50$ for Rosenbock, Ackley, and Rastrigin functions. All results are recorded as the mean of 30 independent runs.

Tables 16, 17, and 18 represent the experimental results of CSSA against NINICSSA, NPARCSSA, and NPOSCSSA on the eighteen UCI datasets, respectively. In general, CSSA outperforms other versions of CSSA in terms of $Mean_{Fit}$, $Mean_{Acc}$, and $Mean_{Feat}$, and it is also clear that CSSA has a significant advantage over NPOSCSSA, winning 16, 11, and 15 times in $Mean_{Fit}$, $Mean_{Acc}$, and $Mean_{Feat}$, respectively. On the other hand, it can be seen that, in terms of $Mean_{Time}$, CSSA has lower computational overhead compared to NINICSSA, NPARCSSA and NPOSCSSACSSA, due to the fact that chaotic map can generate random sequences more simply and efficiently. In short, it is clear that the three improvements proposed in this study are indispensable to boost the overall performance of CSSA, and redefining transgressive position by a chaotic map is especially important.

**Table 16:** Comparison of CSSA and NINICSSA in terms of $Mean_{Fit}$, $Mean_{Acc}$, $Mean_{Feat}$, and $Mean_{Time}$.

Dataset	$Mean_{Fit}$		$Mean_{Acc}$		$Mean_{Feat}$		$Mean_{Time}$	
	CSSA	NINICSSA	CSSA	NINICSSA	CSSA	NINICSSA	CSSA	NINICSSA
BreastCancer	**0.0204**	0.0206	**0.9857**	**0.9857**	**0.6300**	0.6467	3420	**3289**
BreastEW	**0.0367**	**0.0367**	**0.9649**	**0.9649**	**0.1922**	0.2000	**3723**	3975
CongressEW	0.0265	**0.0240**	0.9762	**0.9789**	**0.2938**	0.3188	2379	**2298**
Exactly	**0.0060**	0.0067	**0.9987**	0.9980	**0.4641**	0.4692	5098	**4964**
Exactly2	**0.2211**	0.2232	**0.7815**	0.7797	**0.4795**	0.5026	5173	**4993**
HeartEW	**0.0860**	0.0884	**0.9179**	0.9154	0.4769	**0.4667**	1673	**1650**
IonosphereEW	0.0711	**0.0670**	0.9315	**0.9357**	**0.3245**	0.3333	**3298**	3341
Lymphography	**0.1658**	0.1691	**0.8367**	0.8333	0.4130	**0.4074**	1212	**1192**
WineEW	0.0032	**0.0031**	**1.0000**	**1.0000**	0.3154	**0.3128**	1322	**1303**
Zoo	**0.0033**	**0.0033**	**1.0000**	**1.0000**	**0.3250**	0.3271	1021	**1011**
M-of-n	0.0048	**0.0047**	0.9998	**1.0000**	**0.4667**	**0.4667**	5015	**4983**
PenglungEW	**0.3627**	0.3671	**0.6378**	0.6333	**0.4098**	0.4116	**2196**	2374
SonarEW	0.0188	**0.0165**	0.9849	**0.9873**	0.3906	**0.3889**	**3082**	3179
SpectEW	**0.1087**	0.1100	**0.8938**	0.8926	**0.3636**	0.3667	**1860**	1972
Tic-tac-toe	**0.1552**	0.1560	**0.8531**	0.8521	0.9778	**0.9556**	4572	**4524**
Vote	**0.0024**	**0.0024**	**1.0000**	**1.0000**	0.2396	**0.2375**	1773	**1763**
KrVsKpEW	**0.0256**	**0.0256**	0.9800	**0.9801**	**0.5750**	0.5898	**37344**	38786
WaveformEW	**0.1574**	0.1583	**0.8468**	0.8460	**0.5800**	0.5825	**37761**	38316
Overall	**0.0820**	0.0824	**0.9216**	0.9213	**0.4399**	0.4436	**6773**	6884
W|T|L	**9|4|5**	5|4|9	**8|5|5**	5|5|8	**11|1|6**	6|1|11	7|0|11	**11|0|7**

Significant values are in [bold].

**Table 17:** Comparison of CSSA and NPARCSSA in terms of $Mean_{Fit}$, $Mean_{Acc}$, $Mean_{Feat}$, and $Mean_{Time}$.

Dataset	$Mean_{Fit}$		$Mean_{Acc}$		$Mean_{Feat}$		$Mean_{Time}$	
	CSSA	NPARCSSA	CSSA	NPARCSSA	CSSA	NPARCSSA	CSSA	NPARCSSA
BreastCancer	0.0204	**0.0202**	**0.9857**	**0.9857**	0.6300	**0.6067**	3420	**3272**
BreastEW	**0.0367**	0.0374	**0.9649**	**0.9649**	**0.1922**	0.2689	**3723**	3982
CongressEW	0.0265	**0.0256**	0.9762	**0.9774**	**0.2938**	0.3250	2379	**2330**
Exactly	0.0060	**0.0046**	0.9987	**1.0000**	0.4641	**0.4615**	5098	**4958**
Exactly2	**0.2211**	0.2216	**0.7815**	0.7813	**0.4795**	0.5128	5173	**4988**
HeartEW	0.0860	**0.0832**	0.9179	**0.9210**	**0.4769**	0.5000	1673	**1653**
IonosphereEW	0.0711	**0.0644**	0.9315	**0.9376**	0.3245	**0.2578**	3298	**3284**
Lymphography	**0.1658**	0.1726	**0.8367**	0.8300	**0.4130**	0.4278	1212	**1193**
WineEW	0.0032	**0.0031**	**1.0000**	**1.0000**	0.3154	**0.3077**	1322	**1308**
Zoo	**0.0033**	0.0034	**1.0000**	**1.0000**	**0.3250**	0.3438	1021	**1015**
M-of-n	0.0048	**0.0046**	0.9998	**1.0000**	0.4667	**0.4615**	5015	**4992**
PenglungEW	**0.3627**	0.3848	**0.6378**	0.6156	**0.4098**	0.4172	**2196**	2428
SonarEW	**0.0188**	0.0213	**0.9849**	0.9825	**0.3906**	0.4011	**3082**	3220
SpectEW	**0.1087**	0.1192	**0.8938**	0.8833	**0.3636**	0.3742	**1860**	2007
Tic-tac-toe	**0.1552**	0.1555	**0.8531**	0.8528	0.9778	**0.9704**	4572	**4473**
Vote	**0.0024**	0.0028	**1.0000**	**1.0000**	**0.2396**	0.2792	**1773**	1777
KrVsKpEW	**0.0256**	0.0288	**0.9800**	0.9771	**0.5750**	0.6102	**37344**	38159
WaveformEW	0.1574	**0.1570**	0.8468	**0.8472**	0.5800	**0.5733**	37761	**37240**
Overall	**0.0820**	0.0839	**0.9216**	0.9198	**0.4399**	0.4500	**6773**	6793
W|T|L	**10|0|8**	8|0|10	**7|5|6**	6|5|7	**11|0|7**	7|0|11	6|0|12	**12|0|6**

Significant values are in [bold].

**Table 18:** Comparison of CSSA and NPOCSSA in terms of $Mean_{Fit}$, $Mean_{Acc}$, $Mean_{Feat}$, and $Mean_{Time}$.

Dataset	$Mean_{Fit}$		$Mean_{Acc}$		$Mean_{Feat}$		$Mean_{Time}$	
	CSSA	NPOSCSSA	CSSA	NPOSCSSA	CSSA	NPOSCSSA	CSSA	NPOSCSSA
BreastCancer	**0.0204**	0.0208	**0.9857**	0.9855	**0.6300**	0.6400	3420	**3207**
BreastEW	**0.0367**	0.0372	**0.9649**	**0.9649**	**0.1922**	0.2433	**3723**	3899
CongressEW	0.0265	**0.0244**	0.9762	**0.9785**	**0.2938**	0.3188	2379	**2324**
Exactly	**0.0060**	0.0087	**0.9987**	0.9960	**0.4641**	0.4744	5098	**4848**
Exactly2	**0.2211**	0.2273	**0.7815**	0.7752	0.4795	**0.4718**	5173	**4981**
HeartEW	**0.0860**	0.0890	**0.9179**	0.9148	0.4769	**0.4667**	1673	**1651**
IonosphereEW	**0.0711**	0.0776	**0.9315**	0.9249	**0.3245**	0.3255	**3298**	3532
Lymphography	**0.1658**	0.1718	**0.8367**	0.8311	**0.4130**	0.4556	1212	**1178**
WineEW	**0.0032**	0.0033	**1.0000**	**1.0000**	**0.3154**	0.3333	1322	**1296**
Zoo	**0.0033**	0.0034	**1.0000**	**1.0000**	**0.3250**	0.3417	1021	**997**
M-of-n	**0.0048**	0.0056	**0.9998**	0.9992	**0.4667**	0.4744	5015	**4916**
PenglungEW	**0.3627**	0.3758	**0.6378**	0.6244	0.4098	**0.4019**	**2196**	2397
SonarEW	**0.0188**	0.0213	**0.9849**	0.9825	**0.3906**	0.3978	**3082**	3187
SpectEW	**0.1087**	0.1198	**0.8938**	0.8827	**0.3636**	0.3652	**1860**	2006
Tic-tac-toe	0.1552	**0.1546**	0.8531	**0.8538**	**0.9778**	0.9926	4572	**4279**
Vote	**0.0024**	0.0029	**1.0000**	**1.0000**	**0.2396**	0.2896	1773	**1762**
KrVsKpEW	**0.0256**	0.0258	**0.9800**	**0.9800**	**0.5750**	0.6000	**37344**	38350
WaveformEW	**0.1574**	0.1581	**0.8468**	0.8462	**0.5800**	0.5850	**37761**	41030
Overall	**0.0820**	0.0849	**0.9216**	0.9189	**0.4399**	0.4543	**6773**	6991
W|T|L	**16|0|2**	2|0|16	**11|5|2**	2|5|11	**15|0|3**	3|0|15	7|0|11	**11|0|7**

Significant values are in [bold].

Furthermore, we study exploration merits added to CSSA thanks to its main components. We therefore take the average distance from the swarm center for all sparrows as a measurement of swarm diversity^79^ as

17 $$\begin{aligned} {\mathscr {D}}=\frac{1}{N} \sum _{i=1}^{N} \sqrt{\sum _{j=1}^{D}\left( x_{i,j}-\dot{{\textbf{x}}}_{j}\right) ^{2}}, \end{aligned}$$

where $\dot{{\textbf{x}}}_{j}$ is the value at the *j*-th dimension of the swarm center $\dot{{\textbf{x}}}$. A larger ${\mathscr {D}}$ indicates that the greater the dispersion of individuals in the swarm the higher the swarm diversity, and conversely, the lower the swarm diversity.

**Figure 8:**
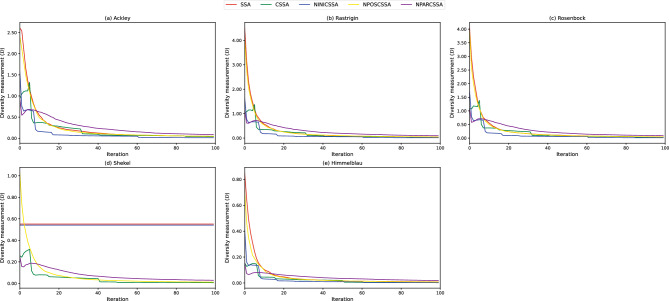
Swarm diversity curves.

Consequently, Fig. 8 compares CSSA with its ablated variants in terms of swarm diversity. As the algorithm gradually converges, individuals reach a similar state, leading to a convergence of the swarm to the minimum as the iterations proceed^79^. It is obvious from Fig. 8 that SSA and NINICSSA always maintain the same swarm diversity on the Shekel function, indicating that the algorithm does not evolve and falls into a local optimum, while the other CSSA variants with chaotic initial swarm gradually converges, showing that initializing the swarm by a chaotic map facilitates the algorithm to jump out of the local optimum. The diversity curves of the remaining functions show that the diversity of NPOSCSSA remains basically the same as that of SSA, and it can be seen that swarm diversity of NPOSCSSA and SSA is high due to the presence of transgressive individuals. However, NPOSCSSA still has its own advantages over SSA. For example, NPOSCSSA converges normally on the Shekel function, indicating that although no updates are made to transgressive sparrows in this version, NPOSCSSA is still able to utilize chaotic maps in the initial swarm and random parameters to enable CSSA to escape from local optima. On the other hand, swarm diversity of NPARCSSA converged smoothly to the minimum point similarly to SSA. It is possible that, like SSA for the Shekel function, a similar situation occurs when NPARCSSA deals with more complex functions, but it is only because NPARCSSA retains chaotic initial swarm and chaotic position updates, i.e., it cannot thus find its deficiencies when the type of function being optimized is limited. In contrast, there is a clear trend in swarm diversity for CSSA when the initial swarm, transgression location, and random parameters are all amended by chaotic maps. In summary, each single improvement embedded into CSSA has its own merit and is indispensable for swarm diversity and avoidance of falling into local optima.

**Figure 9:**
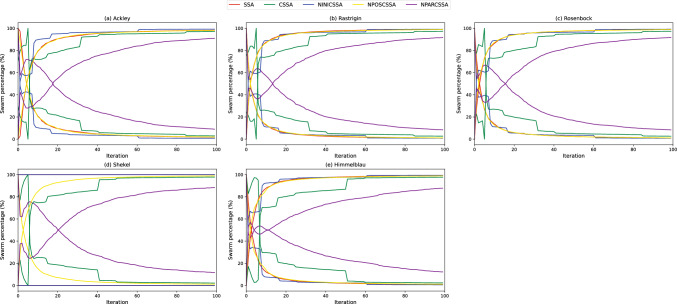
Exploration-exploitation trade-off curves.

From Fig. 9 CSSA have the ability of high exploration and low exploitation, so as to initially explore the solution space comprehensively, and as the iteration increases, the exploration ability of the algorithm gradually diminishes whereas the exploitation ability increase, so as to converge to the global optimal solution more quickly. As can be seen, the exploratory capability of all algorithms except CSSA in the initial phase of all five benchmark functions decreases sharply while the exploitation capability increases sharply. On the contrary, CSSA is able to maintain a decent trade-off by preserving high exploration capability in the initial stage and exploitation capability later, enabling the algorithm to explore the solution space more fully and search feasible regions to find the global optimal solution.

Overall, Figs. 8 and 9 show that: (i) NPOSCSSA has similar performance to SSA but has the ability to avoid local optima, as shown in the test results of the Ackley and Shekel functions; (ii) NINICSSA has a risk of premature convergence but its convergence trend is fluctuating; (iii) NPARCSSA has a smooth convergence trend like SSA, which leads to the risk of the algorithm falling into a local optimum when dealing with more complex problems; and (iv) CSSA retains the above advantages while avoiding the shortcomings, allowing the algorithm to show the best results in terms of swarm diversity, and the balance between exploration and exploitation capabilities.

### CSSA vs. other state-of-the-art optimizers in the literature

Table 19 compares CSSA with other algorithms in the literature, including hybrid evolutionary population dynamics and GOA (BGOA-EPD-Tour)^80^, hybrid gravitational search algorithm (HGSA)^81^, improved HHO (IHHO)^82^, a self-adaptive quantum equilibrium optimizer with ABC (SQEOABC)^83^, binary coyote optimization algorithm (BCOA)^84^, chaotic binary group search optimizer (CGSO5)^85^, and chaos embed marine predator algorithm (CMPA)^86^.

In order to verify whether CSSA has a competitive advantage over similar algorithms, two recently proposed chaotic algorithms, i.e., CGSO5 and CMPA, are chosen among compared algorithms. From Table 19, $Mean_{Acc}$ of CSSA is higher than that of CGSO5 and CMPA on all datasets, except for the CongressEW dataset where it is inferior to CMPA. In addition, the comparison results with other non-chaotic algorithms also show that CSSA has outstanding advantages. In a summary, a comparison with FS literature works demonstrates usefulness and superiority of CSSA over other several, state-of-the-art methods.

**Table 19:** $Mean_{Acc}$ of CSSA compared to other optimizers in the literature.

Dataset	CSSA	BGOA-EPD-Tour	HGSA	IHHO	SQEOABC	CGSO5	CMPA	BCOA
BreastCancer	**0.9857**	0.9800	0.9740	0.9341	0.97429	0.9590	0.9600	0.9632
BreastEW	0.9649	0.9470	**0.9710**	0.9114	0.93721	0.9690	0.9400	−
CongressEW	0.9762	0.9640	0.9660	0.9483	0.9679	0.9710	**0.9800**	−
Exactly	0.9987	0.9990	**1.0000**	0.7167	**1.0000**	0.9740	0.8900	−
Exactly2	0.7815	0.7800	0.7700	**0.7940**	0.7447	0.7840	0.7800	−
HeartEW	**0.9179**	0.8330	0.8560	−	0.8059	0.8320	0.8200	−
IonosphereEW	0.9315	0.8990	0.9340	0.8164	**0.9451**	0.9200	0.9300	−
Lymphography	0.8367	0.8680	**0.8920**	0.7922	0.8847	−	0.8700	−
WineEW	**1.0000**	0.9890	0.9890	−	0.9775	−	0.9700	−
Zoo	**1.0000**	0.9930	0.9320	0.9889	0.9608	0.9630	0.9800	0.9724
M-of-n	0.9998	**1.0000**	**1.0000**	0.9900	**1.0000**	0.9560	−	−
PenglungEW	0.6378	0.9270	0.9560	−	**0.9946**	0.9220	0.9700	−
SonarEW	**0.9849**	0.9120	0.9580	0.6968	0.9064	0.9400	0.900	0.8389
SpectEW	0.8938	0.8260	**0.9190**	−	0.8741	0.8540	0.8300	0.8683
Tic-tac-toe	**0.8531**	0.8080	0.7880	0.7885	0.7829	0.8120	0.7800	−
Vote	**1.0000**	0.9660	0.9730	0.9228	0.9793	0.9700	0.9700	−
KrVsKpEW	0.9800	0.9680	0.9780	0.9348	**0.9817**	0.9530	0.9700	−
WaveformEW	**0.8468**	0.7370	0.8150	0.7764	0.8067	0.8020	0.7900	−
W|T|L	**8|0|10**	0|1|17	3|2|13	1|0|13	3|2|13	0|0|16	1|0|16	0|0|4

Significant values are in [bold].

### CSSA on high-dimensional microarray datasets: The additional experiment

To verify the scalability and robustness of CSSA to tackle FS problems, we further test three high-dimensional microarray datasets having up to 12000 features, namely, 11_Tumors, Brain_Tumor2 and Leukemia2. They are all of high feature size, low sample size, as reported in Table 21. Since high-dimensional data can cause significant time overhead, we prefer to use the experimental settings in Table 20. Tables 22, 23, 24, and 25 show the experimental results in terms of $Mean_{Fit}$, $Mean_{Acc}$, $Mean_{Feat}$, and $Mean_{Time}$, respectively. It is evident that CSSA has outstanding advantages over other algorithms in terms of $Mean_{Fit}$ and $Mean_{Acc}$, but its performance in terms of $Mean_{Feat}$ is relatively poor, which can be justified by the high $Mean_{Acc}$ obtained. On the other hand, all algorithms have a huge overhead in terms of $Mean_{Time}$, which is normally caused by the limitations of the wrapper-based methods themselves. This can be improved by combining other methods (e.g., filter-based methods).

**Table 20:** Special settings for high-dimensional data experiments.

Parameter	Value
Operating system	Linux (Rocky 8.5)
CPU	Four Xeon 6240/18 cores 2.6 GHz (72 cores in total)
RAM	512GB
Software	Python 3.9.7^87^

**Table 21:** High-dimensional microarray datasets.

Dataset	No. of features	No. of instances	No. of classes
11_Tumors	12533	174	11
Brain_Tumor2	10367	50	5
Leukemia2	11225	72	3

**Table 22:** Comparison of CSSA against its peers in terms of $Mean_{Fit}$ on high-dimensional datasets.

Dataset	CSSA	SSA	ABC	PSO	BA	WOA	GOA	HHO	BSA	ASO	HGSO	LSHADE	CMAES
11_Tumors	**0.1454**	0.1530	0.1581	0.1823	0.2101	0.1691	0.1710	0.1557	0.1738	0.1878	0.2037	0.1701	0.1857
Brain_Tumor2	**0.0445**	0.0707	0.0612	0.1039	0.1266	0.0742	0.0875	0.0774	0.0808	0.1003	0.1112	0.0907	0.1022
Leukemia2	0.0047	0.0045	0.0049	0.0046	**0.0040**	0.0047	0.0049	0.0046	0.0048	0.0049	0.0050	0.0049	0.0061
Overall	**0.0649**	0.0761	0.0747	0.0969	0.1136	0.0827	0.0878	0.0792	0.0865	0.0977	0.1066	0.0886	0.0980
Friedman rank	2.5	**2.33**	5.17	7.83	9	4.83	7.83	3.83	7	9.83	12	7.83	11
Final rank	2	**1**	5	7	10	4	7	3	6	11	13	7	12

Significant values are in [bold].

**Table 23:** Comparison of CSSA against its peers in terms of $Mean_{Acc}$ on high-dimensional datasets.

Dataset	CSSA	SSA	ABC	PSO	BA	WOA	GOA	HHO	BSA	ASO	HGSO	LSHADE	CMAES
11_Tumors	**0.8581**	0.8505	0.8457	0.8210	0.7924	0.8343	0.8324	0.8476	0.8295	0.8152	0.8000	0.8333	0.8190
Brain_Tumor2	**0.9600**	0.9333	0.9433	0.9000	0.8767	0.9300	0.9167	0.9267	0.9233	0.9033	0.8933	0.9133	0.9033
Leukemia2	**1.0000**	**1.0000**	**1.0000**	**1.0000**	**1.0000**	**1.0000**	**1.0000**	**1.0000**	**1.0000**	**1.0000**	**1.0000**	**1.0000**	**1.0000**
Overall	**0.9394**	0.9279	0.9297	0.9070	0.8897	0.9214	0.9164	0.9248	0.9176	0.9062	0.8978	0.9155	0.9074
Friedman rank	**3**	4	4.33	9	11	5.33	7	5	7	9.17	10.33	7	8.83
Final rank	**1**	2	3	10	13	5	6	4	6	11	12	6	9

Significant values are in [bold].

**Table 24:** Comparison of CSSA against its peers in terms of $Mean_{Feat}$ on high-dimensional datasets.

Dataset	CSSA	SSA	ABC	PSO	BA	WOA	GOA	HHO	BSA	ASO	HGSO	LSHADE	CMAES
11_Tumors	0.4944	0.4946	0.5333	0.5010	**0.4597**	0.5014	0.5060	0.4888	0.5048	0.4881	0.5668	0.5067	0.6553
Brain_Tumor2	0.4863	0.4729	0.5094	0.4854	**0.4462**	0.4889	0.4979	0.4789	0.4946	0.4615	0.5596	0.4942	0.6499
Leukemia2	0.4711	0.4511	0.4940	0.4617	**0.3971**	0.4688	0.4911	0.4637	0.4819	0.4864	0.5027	0.4869	0.6088
Overall	0.4839	0.4729	0.5122	0.4827	**0.4343**	0.4864	0.4983	0.4771	0.4938	0.4787	0.5430	0.4959	0.6380
Friedman rank	5.33	3.33	11	4.67	**1**	6.33	9.67	3.67	8	4	12	9	13
Final rank	6	2	11	5	**1**	7	10	3	8	4	12	9	13

Significant values are in [bold].

**Table 25:** Comparison of CSSA against its peers in terms of $Mean_{Time}$ on high-dimensional datasets.

Dataset	CSSA	SSA	ABC	PSO	BA	WOA	GOA	HHO	BSA	ASO	HGSO	LSHADE	CMAES
11_Tumors	83526	82066	245040	85060	63279	84592	456043	63657	74359	82176	84728	**47081**	13595806
Brain_Tumor2	58936	59218	168041	61423	44598	58373	355852	45270	52848	58053	61458	**33392**	8316136
Leukemia2	65329	65784	187711	68378	49587	65169	386624	50568	57887	60323	68963	**36942**	10039133
Overall	69264	69023	200264	71620	52488	69378	399506	53165	61698	66851	71717	**39138**	10650358
Friedman rank	7	7	11	9.33	2	6.67	12	3	4	5.33	9.67	**1**	13
Final rank	7	7	11	9	2	6	12	3	4	5	10	**1**	13

Significant values are in [bold].

## Discussion

In order to cope with issues encountered in standard SSA, such as early loss of swarm diversity and hence easily falling into local optima, this study integrates chaotic maps into SSA to produce CSSA. The effectiveness of CSSA has been demonstrated through many comparative and analytical studies. The main purpose of this section is to give a brief summary of the strengths and weaknesses of CSSA.

CSSA has the following advantages:

1. The improvement effect of ten chaotic maps on SSA is researched completely in this work, and thus the degree of contribution of diverse chaotic maps is examined from a global perspective. The best CSSA determined in this manner can avoid the one-sidedness of a single chaotic map and serve as a reference for subsequent research.
2. CSSA improves the performance of SSA while reducing its computational cost. From Table 7, it can be seen that CSSA significantly improves the performance of the algorithm in terms of $Mean_{Fit}$, $Mean_{Acc}$, $Mean_{Feat}$, and $Mean_{Time}$ without highly increasing the computational cost.
3. Tables 9, 10, 11, and 12 describe in detail the results of CSSA compared with twelve well-known algorithms in terms of $Mean_{Fit}$, $Mean_{Acc}$, $Mean_{Feat}$, and $Mean_{Time}$. Figures 3, 4, and 5 visualize the classification accuracy and feature reduction rate performance of all competitors. It can be seen that CSSA effectively reduces the $Mean_{Feat}$ (0.4399) while achieving the highest $Mean_{Acc}$ (0.9216). In addition, CSSA’s ability to handle truly high-dimensional data has been demonstrated through experiments on three microarray datasets with up to 12000 features.
4. Furthermore, seven recently proposed methods selected from the literature are compared with CSSA, and the comparative study shows that our proposed method not only outperforms other non-chaotic algorithms but also has outstanding advantages among similar chaotic ones.

In addition, CSSA has its own limitations:

1. Table 12 demonstrates that CSSA is not optimal in terms of $Mean_{Time}$, which may be due to the fact that SSA was originally developed for continuous search space. Although the V-shaped function in Eq. (7) allows CSSA to deal with discrete problems, its essence is still evolving via a continuous approach. As a result, to improve overall performance and reduce computational costs, a more efficient SSA variant for discrete problems can be designed.
2. It is vital to note that CSSA cannot successfully minimize the $Mean_{Feat}$ when dealing with extremely high-dimensional data. Table 24 demonstrates that CSSA picks more than 5000 features (a nearly 50% reduction) on all three datasets, indicating that the algorithm cannot successfully reduce selected feature size and is not conducive to the analysis and extraction of valuable features. This issue can be overcome by combining the filters (which are used to reduce and select high-quality features) and wrappers (which are used to improve the algorithm’s performance). CSSA, on the other hand, achieves superior superior $Mean_{Fit}$ and $Mean_{Acc}$, as seen in Tables 22 and 23, respectively.

## Conclusion

In this paper, a new chaotic sparrow search algorithm (CSSA) is suggested and used to FS problems. The majority of the literature focuses on the influence of a single chaotic map on an algorithm. Ten chaotic maps are investigated in this study comprehensively. Based on our findings, CSSA with Chebyshev and Circle chaotic maps embedded into it delivers the best outcomes among evaluated schemes by making a good trade-off between exploration and exploitation in CSSA. CSSA offers a competitive edge in global optimization and addressing FS problems when compared to twelve state-of-the-art algorithms, including LSHADE and CMAES, and seven recently proposed, relevant approaches in the literature, according to comparative research. Furthermore, a post-hoc statistical analysis confirms CSSA’s significance on most UCI datasets and high-dimensional microarray datasets, demonstrating that CSSA has an exceptional ability to pick favorable features while achieving high classification accuracy.

However, when dealing with high-dimensional datasets, CSSA’s time cost is not satisfactory when compared to its contemporaries, and the feature selection ratio is not successfully reduced. To address these concerns, we propose to integrate the filters and wrappers in future work, in order to leverage their respective benefits in building a new binary SSA version that is more suitable for high-dimensional FS problems.

## Data Availability

The datasets generated and/or analyzed during the current study are available from the corresponding author on reasonable request.
